# CONY: A Bayesian procedure for detecting copy number variations from sequencing read depths

**DOI:** 10.1038/s41598-020-64353-1

**Published:** 2020-06-26

**Authors:** Yu-Chung Wei, Guan-Hua Huang

**Affiliations:** 1 Graduate Institute of Statistics and Information Science, National Changhua University of Education, No.1 Jinde Road, Changhua City, Changhua County 50007 Taiwan; 2 Institute of Statistics, National Chiao Tung University, 1001 University Road, Hsinchu, 30010 Taiwan

**Keywords:** Statistical methods, Next-generation sequencing, Bayesian inference

## Abstract

Copy number variations (CNVs) are genomic structural mutations consisting of abnormal numbers of fragment copies. Next-generation sequencing of read-depth signals mirrors these variants. Some tools used to predict CNVs by depth have been published, but most of these tools can be applied to only a specific data type due to modeling limitations. We develop a tool for **co**py **n**umber variation detection by a Ba**y**esian procedure, i.e., CONY, that adopts a Bayesian hierarchical model and an efficient reversible-jump Markov chain Monte Carlo inference algorithm for whole genome sequencing of read-depth data. CONY can be applied not only to individual samples for estimating the absolute number of copies but also to case-control pairs for detecting patient-specific variations. We evaluate the performance of CONY and compare CONY with competing approaches through simulations and by using experimental data from the 1000 Genomes Project. CONY outperforms the other methods in terms of accuracy in both single-sample and paired-samples analyses. In addition, CONY performs well regardless of whether the data coverage is high or low. CONY is useful for detecting both absolute and relative CNVs from read-depth data sequences. The package is available at https://github.com/weiyuchung/CONY.

## Introduction

Copy number variations (CNVs) are genomic structural mutations consisting of abnormal numbers of deoxyribonucleic acid (DNA) section copies. CNVs were originally defined to range from one kilobasepair to several megabasepairs^1,2^ and widened to include small variants that are larger than 50 basepairs in size^3,4^. Currently, approximately 7 million CNVs identified in 1 million variant regions are catalogued in the Database of Genomic Variants (DGV)^5,6^. Half the identified CNVs overlap with protein-coding regions, which results in gene expression changes^7^. CNVs have been confirmed to play important roles in human diseases; for example, glycophorin CNVs in malaria resistance^8^, beta-defensin CNVs in Psoriasis^9,10^, CNVs in 15q11.2 for the perigenual anterior cingulate cortex in schizophrenia and Alzheimer’s disease^11,12^, and some pathogenic CNVs in developmental delay, autism spectrum disorders, and various congenital malformations^13,14^. Furthermore, somatic copy number aberrations have been considered to be associated with human cancers and to categorize the subtypes of cancer^15^, such as breast cancer^16,17^, lung cancer^18,19^, and colorectal cancer^20,21^.

Array comparative genomic hybridization^22,23^ and single nucleotide polymorphism arrays^24,25^ have been used to detect CNVs over the past few years; however, the boundaries of CNVs cannot be explicitly identified due to the sparse probe coverage. Recently, next-generation sequencing (NGS)^26,27^ has provided a more accurate option for CNV identification and breakpoint prediction through the high-resolution analysis of sequential DNA nucleotide bases. Various strategies, including read depth^28–36^, paired-end mapping^37–40^, split read^41–43^, assembly^44–46^ and integrative approaches^47–51^, have been adopted to detect CNVs in NGS data. Read depth analysis becomes a major method because of less restriction for read lengths and insert sizes^26,27,52^, which are critical limitations for other strategies. Besides, depth data can be derived from both paired- and single-end sequencing reads with appropriate mapping and normalizing procedures.

In the read-depth approach, CNV identification assumes that the number of reads is proportional to the number of DNA copies. Hypothesis testing, change point segmentation, and the hidden Markov model are commonly used methods in this field. While many practical tools have been developed using these types of statistical algorithms, the link between sequencing depth information and CNVs is not completely understood. In hypothesis testing methods, each depth is independently tested for a significant CNV^35,36^, but correlation of depths should be considered through the corresponding genomic locations. The adjustment methods used for multiple testing issues also need to be evaluated rigorously. In change point algorithms, copy number (CN) regions are first identified by a segmentation algorithm, and then the states of the proposed CN regions are estimated^30^. However, the performance of the segmentation step has an obvious impact on the downstream CNV detection accuracy. To overcome these shortcomings, a statistical model approach that considers genetic information from whole genome sequencing depths to simultaneously identify CN regions and states is presented in this paper.

In addition, most existing approaches were proposed for a specific sample design. Single-sample analyses can estimate absolute CN callings^28,29,36^ and are implemented in personalized medicine^53,54^. However, read data from one single sample only contain individual genomic information, not population-level variations; as a result, it is not easy to find the potential biases especially in the low coverage data. In contrast, depth ratios of paired samples (case/control or tumor/normal) identify patient-specific relative CNVs and are conveniently utilized in association studies^29,35^. While background or platform noises may be efficiently eliminated through the comparative depths, combining sample information from different sequencing coverages or platforms remain difficult issues. The proposed model-based algorithm in this study could be applied to various sample designs due to the thorough data transformation and the parameter settings.

Given the aforementioned challenges, we propose a comprehensive approach called **co**py **n**umber variation detection via a Ba**y**esian procedure (CONY). A Bayesian hierarchical model is constructed to integrate the sequencing depth signals, the corresponding genomic position, and the potential CNV information. The efficient sampling algorithm, i.e., reversible-jump Markov chain Monte Carlo (RJMCMC)^55^, is modified to infer the states and breakpoints of the CN regions. An appropriate analytic section length of the genome for the RJMCMC algorithm is suggested to reduce the unbalanced effects that result from the extreme difference between normal and variant region sizes. The usefulness of the CONY algorithm is demonstrated by both simulations and an analysis of experimental data from the 1000 Genomes Project^56^.

## Materials

### The 1000 Genomes Project

Whole genome sequencing data of two samples NA12156 and NA12878 (SRA accessions ERX000125 and ERX000080, respectively) provided by the 1000 Genomes Project were analyzed. Each of the samples was used to identify the absolute CNVs, and they were matched to form case/control pairs (NA12156/NA12878 and NA12878/NA12156) to identify the case-specific relative CNVs. The identified CNVs were compared with CNV lists reported in the Database of Genomic Variants^5,6^. Sequencing reads generated by the Illumina platform with 4.1 to 5.7X coverage and mapped to the human genome 19 (hg19/GRCh37) reference genome with default adjustments were downloaded from the 1000 Genomes Project ftp.

Another two experimental samples HG00419 and HG01595 from the project, which were sequenced with both low (5.2 to 9.8X as SRA accessions SRX724413 and SRX720422, respectively) and high (33.6 to 35.4X as SRA accessions SRX550074 and SRX550114, respectively) coverages, were also analyzed to show consistency of results from CONY across samples and evaluate the coverage effect. Both samples were used for the single-sample analysis; HG00419/HG01595 and HG01595/HG00419 were matched for the paired-samples analysis. Reads mapped to the hg38/GRCh38 human reference genomes were adopted.

### The simulation study

In the single-sample analysis, DNA sequences were generated from one hundred samples with predetermined CNVs. We used the hg19 chromosome 20 (chr20) as the template. The template sequence was copied to one strand and deleted/duplicated in pieces to mimic the copy loss/gain to the other strand for each sample. Twenty pieces for copy losses were deleted from the variant strand as copy number (CN) 1, and twenty pieces for copy gains were randomly duplicated 1, 2, or 3 times as CN 3, 4, or 5, respectively. The artificial pieces were set at 10 different sizes (1, 2.5, 5, 10, 25, 50, 100, 250, 500, and 1000 kilobasepairs (kb)) using 2 of each for the copy losses/gains. The synthetic CNV regions accounted for 12% of the human genome, which is consistent with a recent report^1,7^. In the paired-samples analysis, simulated samples from the single-sample analysis were used as case samples. One common control sample sequence was copied from the hg19 chr20 template for both strands. In total, two million paired-end reads with a length of 70 basepairs (bp) and a coverage of 2.2X (low coverage) or 22X (high coverage) were generated for each sample via the sequencing simulation software Wgsim^57^. The simulated reads were aligned to the reference genome by BWA^58^ and subjected to data preprocessing.

## Methods

A Bayesian model-based procedure, i.e., CONY, that is able to identify both absolute and relative CNVs from both single-sample and paired-samples DNA sequencing is proposed. In this procedure, read-depth signals (RDSs) derived from preprocessed sequencing reads are used to estimate CNVs via a Bayesian hierarchical model and the RJMCMC algorithm.

The sequencing reads are aligned with the reference genome, subjected to preprocessing steps, and accumulated as read depths per base via published tools^59^. Next, the base-read depths in a small contiguous region (referred to as a window) are summed as the window read depth of each sample. After adjusting for potential biases, the window read depths are transformed to RDSs by logarithm (single-sample analysis) or log-ratio (paired-samples analysis) equations.

**Figure 1:**
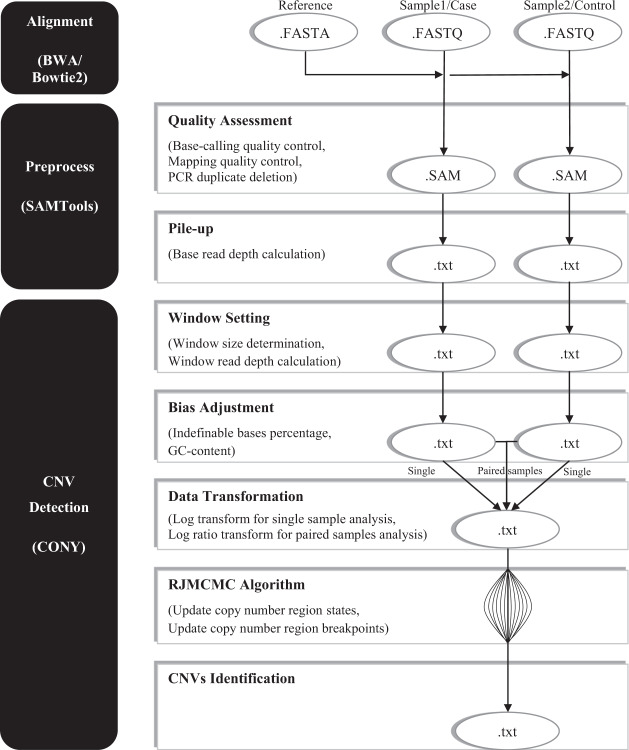
Flowchart of read alignment, data preprocessing and CNV detection.

RDSs are linked to the states and breakpoints of CN regions via a comprehensive Bayesian hierarchical model. A modified RJMCMC algorithm is constructed to generate samples for parameter inferences with two main moves (updating CN states and updating boundaries) and four jumping strategies (merge, split, trifid, and boundary change) for updating the boundaries. After 5,000 burn-ins, the windows with the abnormal CNs are tested via Bayes factors in each additional 1,000 iterations until full coverage is achieved. The details of the CONY procedure are provided in the following discussion, and a flow chart of the analysis is depicted in Fig. 1.

### Read alignment, data preprocessing, and read-depth signal calculation

First, the decoded sample sequencing reads (FASTQ format) are aligned with the reference sequence (FASTA file) to ensure the corresponding locations in the genome via commonly used tools, such as BWA^58^ and Bowtie2^59^. The best-matched position information of each read is written in SAM/BAM format using SAMtools software. The low-quality reads and experimental duplicates are removed, including base-calling quality scores lower than 13, mapping quality scores lower than 30, and PCR duplicates^36,60,61^. Then, the good-quality reads are piled to obtain accumulated measurements of each nucleotide, which are referred to as the “base-read depth”.

Base-read depths are insufficient for identifying CNVs with high specificity^47^. The potential systematic biases easily override the true CNV evidence because of the weak information from a single base. Moreover, a single signal has insufficient statistical strength to support the assumption of a uniform relationship between the CNVs and read depth. To increase the power of the read-depth information, the summarized signals from several bases are considered. A series of consecutive bases constitute a window, and the depths of the bases within the window are accumulated to obtain stable and convincing read-depth information^35,36^. Generally, the genome is partitioned into nonoverlapping sliding windows with an equal size of 100 bp as a default^28,36,62^, and the base-read depths in each window are summed as the raw “window read depth”. The *i*^th^ raw window read depth is denoted by *R*_*Raw,i*_.

Two major bias effects (i.e., the percentage of indefinable bases and GC content) should be adjusted to strengthen the evidence of CNVs in the raw window read depths^63,64^. First, the percentage of bases with N code (i.e., indefinable bases) should be considered. Because no depths are counted for these indefinable bases, the window read depths should be adjusted to balance the information across windows. Then, the *i*^th^ window read depth is adjusted by the following equation: ${R}_{CorrSize,i}={R}_{Raw,i}$ × (window size)/(window size -number of indefinable bases in the *i*^th^ window). Second, the GC content is a notable source of noise in the depth estimation, especially using the Illumina platform^63^. The method used for the GC content adjustment follows that of a published study^65,66^. The GC-adjusted window read depth is calculated by the following formula if the percentage of G and C codes in the *i*^th^ window is in the range from 20% to 80%: ${R}_{CorrGC,i}={R}_{CorrSize,i}\times \overline{{R}_{GC}}/{R}_{GC,i}$, where *R*_*GC,i*_ and $\overline{{R}_{GC}}$ represent the predicted depth in the *i*^th^ window via a local regression (LOESS) and the average depth over all windows. Regarding the LOESS model settings, the proportion of neighborhood points is spanned to 75%, and the weight follows a typical tri-cubic function. Since the LOESS adjustment does not work for extreme GC percentages (<20% or>80%), the depths of these windows are not adjusted; thus, ${R}_{CorrGC,i}={R}_{CorrSize,i}$. Furthermore, windows with more than half indefinable codes or zero depth are marked and excluded from further analysis. Then, the window read-depth signals (hereafter referred to as RDSs, *D*_*i*_) for the single-sample and paired-samples analyses are calculated by logarithm (i.e., ${D}_{i}=\,\log ({R}_{CorrGC,i})$) and log-ratio (i.e., ${D}_{i}=\,\log ({R}_{CorrGC,i({\rm{Case}})}/{R}_{CorrGC,i(Control)})$) transformations.

### Bayesian hierarchical model

Following the process outlined above, the adjusted window RDSs were prepared for a downtrend application to estimate the CNVs. A Bayesian hierarchical model is proposed for detecting the absolute/relative CNVs from single-sample/paired-samples window RDSs.

**Figure 2:**
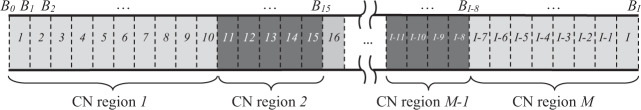
Windows and copy number (CN) regions. The genome is partitioned into *I* sliding, nonoverlapping, equally sized windows as box symbols with RDSs of ${D}_{1},{D}_{2},\ldots ,{D}_{I}$ and boundaries of ${B}_{0},{B}_{1},\ldots ,{B}_{I}$ shown as dashed lines. The Bayesian model aims to group the *I* windows into *M* CN regions with states ${C}_{1}^{s},{C}_{2}^{s},\ldots ,{C}_{M}^{s}$ as box colors that are determined by ${\mathop{{\bf{C}}}\limits_{\sim}}_{1},{\mathop{{\bf{C}}}\limits_{\sim}}_{2},\ldots ,{\mathop{{\bf{C}}}\limits_{\sim}}_{M}$.

This model aims to divide the whole genome with *I* windows into *M* CN regions to group consecutive windows with the same underlying CN. The comprehensive structure constructs the relationships among the window RDSs ($$\mathop{{\bf{D}}}\limits_{\sim}=[{D}_{1},{D}_{2},\ldots ,{D}_{I}]$$), CN states underlying CN regions ($$\mathop{{\bf{C}}}\limits_{\sim}=[{\mathop{{\bf{C}}}\limits_{\sim}}_{1},{\mathop{{\bf{C}}}\limits_{\sim}}_{2},\ldots ,{\mathop{{\bf{C}}}\limits_{\sim}}_{M}]$$) and CN breakpoint indicators of window boundaries ($$\mathop{{\bf{B}}\hspace{0.0005pt}}\limits_{\sim}=[{B}_{0},{B}_{1},\ldots ,{B}_{I}]$$) (Fig. 2). The Bayesian approach starts with the prior belief that the parameters follow the prior distribution $$p(\mathop{{\bf{B}}}\limits_{\sim},\mathop{{\bf{C}}}\limits_{\sim})$$ and uses the likelihood from data $$p(\mathop{{\bf{D}}}\limits_{\sim}|\mathop{{\bf{B}}\hspace{0.0005pt}}\limits_{\sim},\mathop{{\bf{C}}}\limits_{\sim})$$ to update the parameters a posterior. Unlike some existing tools, our proposed Bayesian hierarchical model comprehensively considers parameter relations across the analytic genome rather than just information in consecutive windows. The inferences are based on the posterior distribution $$p(\mathop{{\bf{B}}\hspace{0.0005pt}}\limits_{\sim},\mathop{{\bf{C}}}\limits_{\sim}|\mathop{{\bf{D}}}\limits_{\sim})$$, which is proportional to the priors multiplying the data likelihood, $$p(\mathop{{\bf{B}}\hspace{0.0005pt}}\limits_{\sim},\mathop{{\bf{C}}}\limits_{\sim}|\mathop{{\bf{D}}}\limits_{\sim})\propto p(\mathop{{\bf{B}}\hspace{0.0005pt}}\limits_{\sim},\mathop{{\bf{C}}}\limits_{\sim})\times p(\mathop{{\bf{D}}}\limits_{\sim}|\mathop{{\bf{B}}\hspace{0.0005pt}}\limits_{\sim},\mathop{{\bf{C}}}\limits_{\sim})$$$$\,=\,p(\mathop{{\bf{B}}\hspace{0.0005pt}}\limits_{\sim})\times p(\mathop{{\bf{C}}}\limits_{\sim}|\mathop{{\bf{B}}\hspace{0.0005pt}}\limits_{\sim})\times p(\mathop{{\bf{D}}}\limits_{\sim}|\mathop{{\bf{B}}\hspace{0.0005pt}}\limits_{\sim},\mathop{{\bf{C}}}\limits_{\sim})$$. Three parts are included in factorization, including the window boundary ($$p(\mathop{{\bf{B}}\hspace{0.0005pt}}\limits_{\sim})$$), CN state ($$p(\mathop{{\bf{C}}}\limits_{\sim}|\mathop{{\bf{B}}\hspace{0.0005pt}}\limits_{\sim})$$), and depth ($$p(\mathop{{\bf{D}}}\limits_{\sim}|\mathop{{\bf{B}}\hspace{0.0005pt}}\limits_{\sim},\mathop{{\bf{C}}}\limits_{\sim})$$). The details of factorization and the hyperparameter settings are shown below.

### Window boundary part ${\boldsymbol{p}}(\mathop{{\bf{B}}\hspace{0.0005pt}}\limits_{\sim})$

Parameter $$\mathop{{\bf{B}}}\limits_{\sim}=[{B}_{0},{B}_{1},\ldots ,{B}_{I}]$$ is used to represent whether the window boundaries are the breakpoints of the CN regions. *B*_*i*_ is 1 if windows *i* and *i* + 1 have different underlying CNs for $$i=1,2,\mathrm{..}.,I-1$$ (i.e., the *i*^th^ window boundary is the breakpoint of two CN regions). Otherwise, *B*_*i*_ is denoted by 0. *B*_0_ and *B*_*I*_ are set to 1 due to the left and right borders. Assume that *B*_*i*_ follows an independent Bernoulli distribution with success probability *λ*. The probability of the window boundaries is $$p(\mathop{{\bf{B}}\hspace{0.0005pt}}\limits_{\sim})=p({B}_{0},{B}_{1},\ldots ,{B}_{I})=$$$${\lambda }^{M-1}\times {(1-\lambda )}^{I-M}$$, where $$M={\sum }_{i=1}^{I}{B}_{i}$$. Thus, there is a quantity *M* of *B*_*i*_ with a value of 1 for *i* from 1 to *I*, and the genome is separated into *M* CN regions.

### Copy number state part ${\boldsymbol{p}}(\mathop{{\bf{C}}}\limits_{{\boldsymbol{ \sim }}}|\mathop{{\bf{B}}\hspace{0.0005pt}}\limits_{\sim})$

The whole genome is divided into *M* CN regions when breakpoints $$\mathop{{\bf{B}}\hspace{0.0005pt}}\limits_{\sim}$$ are given. Next, the CN states of each region ($$\mathop{{\bf{C}}}\limits_{\sim}=[{\mathop{{\bf{C}}}\limits_{\sim}}_{1},{\mathop{{\bf{C}}}\limits_{\sim}}_{2},\ldots ,{\mathop{{\bf{C}}}\limits_{\sim}}_{M}]$$) are described based on conditional probability $$p(\mathop{{\bf{C}}}\limits_{\sim}|\mathop{{\bf{B}}\hspace{0.0005pt}}\limits_{\sim})$$$$=\,p({\mathop{{\bf{C}}}\limits_{ \sim }}_{1},...,{\mathop{{\bf{C}}}\limits_{ \sim }}_{M}|\mathop{{\bf{B}}\hspace{0.0005pt}}\limits_{\sim})$$, which can be factorized as $$p({\mathop{{\bf{C}}}\limits_{ \sim }}_{1}|\mathop{{\bf{B}}\hspace{0.0005pt}}\limits_{\sim})\times p({\mathop{{\bf{C}}}\limits_{ \sim }}_{2}|{\mathop{{\bf{C}}}\limits_{ \sim }}_{1},\mathop{{\bf{B}}\hspace{0.0005pt}}\limits_{\sim})\times \ldots \times $$$$p({\mathop{{\bf{C}}}\limits_{\sim}}_{M}|{\mathop{{\bf{C}}}\limits_{\sim}}_{1},\mathrm{..}.,{\mathop{{\bf{C}}}\limits_{\sim}}_{M-1},\mathop{{\bf{B}}\hspace{0.0005pt}}\limits_{\sim})$$. Because the consecutive CN regions must have different states, the state of each region is restricted to the adjacent sides. Therefore, the conditional probability is simplified as $$p({\mathop{{\bf{C}}}\limits_{\sim}}_{1}|\mathop{{\bf{B}}\hspace{0.0005pt}}\limits_{\sim})\times p({\mathop{{\bf{C}}}\limits_{\sim}}_{2}|{\mathop{{\bf{C}}}\limits_{\sim}}_{1},\mathop{{\bf{B}}\hspace{0.0005pt}}\limits_{\sim})\times \ldots \times p({\mathop{{\bf{C}}}\limits_{\sim}}_{M}|{\mathop{{\bf{C}}}\limits_{\sim}}_{M-1},\mathop{{\bf{B}}\hspace{0.0005pt}}\limits_{\sim})$$.

For the state of the first region ${\mathop{{\bf{C}}}\limits_{\sim}}_{1}=[{C}_{11},{C}_{12},\ldots ,{C}_{1K}]$, a one-trial multinomial distribution with a prespecified category number *K* is adopted, i.e., $[{C}_{11},{C}_{12},\cdots ,{C}_{1K}] \sim Multinomial(1;{W}_{F1},{W}_{F2},\cdots ,{W}_{FK})$. If the element ${C}_{1{C}_{1}^{s}}$ of ${\mathop{{\bf{C}}}\limits_{\sim}}_{1}$ is equal to 1, then the CN state of the first region is denoted by ${C}_{1}^{s}$.The weight vector ${\mathop{{\bf{W}}}\limits_{\sim}}_{F}=[{W}_{F1},{W}_{F2},\ldots ,{W}_{FK}]$ follows a conjugate Dirichlet distribution with hyperparameter ${\mathop{{\bf{W}}}\limits_{\sim}}_{{\bf{0}}}=[{w}_{01},{w}_{02},\ldots ,{w}_{0K}]$.

The state of the other regions must be different from the previous state based on the above conditional probability factorization. Assuming the state of the (*m − 1*)^th^ region is *k* (i.e., ${C}_{(m-1)k}=1$ or ${C}_{m-1}^{s}=k$), the state of the *m*^th^ region ${\mathop{{\bf{C}}}\limits_{\sim}}_{m}=[{C}_{m1},{C}_{m2},\cdots ,{C}_{m(k-1)},{C}_{m(k+1)},\cdots ,{C}_{mK}]$ could decrease by one dimension with *K*−1 categories. ${\mathop{{\bf{C}}}\limits_{\sim}}_{m}$ follows a one-trial multinomial distribution with weight vector ${\mathop{{\bf{W}}}\limits_{\sim}}_{k}=[{W}_{k1},{W}_{k2},\ldots ,{W}_{k(k-1)},{W}_{k(k+1)},\ldots ,{W}_{mK}]$, and the weight is Dirichlet distributed with parameter $[\frac{{w}_{01}}{1-{w}_{0k}},\frac{{w}_{02}}{1-{w}_{0k}},\cdots ,\frac{{w}_{0(k-1)}}{1-{w}_{0k}},\frac{{w}_{0(k+1)}}{1-{w}_{0k}},\cdots ,\frac{{w}_{0K}}{1-{w}_{0k}}]$. The hyperparameter ${\mathop{{\bf{W}}}\limits_{\sim}}_{{\bf{0}}}=[{w}_{01},{w}_{02},\ldots ,{w}_{0K}]$ of weight $\mathop{{\bf{W}}}\limits_{\sim}=[{\mathop{{\bf{W}}}\limits_{\sim}}_{F},{\mathop{{\bf{W}}}\limits_{\sim}}_{1},{\mathop{{\bf{W}}}\limits_{\sim}}_{2},\ldots ,{\mathop{{\bf{W}}}\limits_{\sim}}_{K}]$ is estimated via the empirical method introduced in Supplementary Text 1 (Hyperparameters setting).

The conditional probability of the CN states given the breakpoints is summarized as follows:

$$\begin{array}{l}P(\mathop{{\bf{C}}}\limits_{\sim}|\mathop{{\bf{B}}\hspace{0.0005pt}}\limits_{\sim})=\int p({\mathop{{\bf{C}}}\limits_{\sim}}_{1}|\mathop{{\bf{B}}\hspace{0.0005pt}}\limits_{\sim},\mathop{{\bf{W}}}\limits_{\sim})\times p({\mathop{{\bf{C}}}\limits_{\sim}}_{2}|{\mathop{{\bf{C}}}\limits_{\sim}}_{1},\mathop{{\bf{B}}\hspace{0.0005pt}}\limits_{\sim},\mathop{{\bf{W}}}\limits_{\sim})\times \cdots \times p({\mathop{{\bf{C}}}\limits_{\sim}}_{M}|{\mathop{{\bf{C}}}\limits_{\sim}}_{M-1},\mathop{{\bf{B}}\hspace{0.0005pt}}\limits_{\sim},\mathop{{\bf{W}}}\limits_{\sim})\times P(\mathop{{\bf{W}}}\limits_{\sim}|\mathop{{\bf{B}}}\limits_{\sim})d\mathop{{\bf{W}}}\limits_{\sim}\\ \,=\,\{\mathop{\prod }\limits_{k=1}^{K}\frac{\varGamma ({C}_{1k}+{w}_{0k})}{\varGamma ({w}_{0k})}\}\times \mathop{\prod }\limits_{k\text{'}=1}^{K}\{\frac{1}{\varGamma (1+{n}_{k\text{'}})}\mathop{\prod }\limits_{\begin{array}{c}k=1\\ k\ne k\text{'}\end{array}}^{K}\frac{\varGamma ({n}_{k\text{'}k}+\frac{{w}_{0k}}{1-{w}_{0k\text{'}}})}{\varGamma (\frac{{w}_{0k}}{1-{w}_{0k\text{'}}})}\}\end{array}$$

where ${n}_{k\text{'}}$ is the number of regions located after the regions with CN state *k*′, and ${n}_{k\text{'}k}$ is the number of regions with state *k* among these ${n}_{k\text{'}}$ regions. Based on this formula, this model connects information not only from these regions with identical CN states but also from the same previous regions to strengthen the state relationship.

In addition, the number of state categories *K* needs to be pre-assigned in this procedure. For a single-sample analysis, the states represent the absolute CN, and we set *K* = 5 as the default. For paired samples, the states represent the relative CN, and we set *K* = 3 as the default, representing copy loss, normal and copy gain statuses.

### Depth part $p(\mathop{{\bf{D}}}\limits_{\sim}|\mathop{{\bf{B}}\hspace{0.0005pt}}\limits_{\sim},\mathop{{\bf{C}}}\limits_{\sim})$

Given the breakpoints and states of each CN region, we assume that RDSs within the same copy number region follow an independent normal distribution with a common mean and variance. Moreover, the normal and inverse-gamma conjugate priors connect windows from different CN regions that belong to the same CN state. Therefore, the conditional likelihood is derived as follows:

$$p(\mathop{{\bf{D}}}\limits_{\sim}|\mathop{{\bf{B}}\hspace{0.0005pt}}\limits_{\sim},\mathop{{\bf{C}}}\limits_{\sim})$$

$$=p({D}_{1}|\mathop{{\bf{C}}}\limits_{\sim},\mathop{{\bf{B}}\hspace{0.0005pt}}\limits_{\sim})\times p({D}_{2}|\mathop{{\bf{C}}}\limits_{\sim},\mathop{{\bf{B}}\hspace{0.0005pt}}\limits_{\sim})\times \cdots \times p({D}_{I}|\mathop{{\bf{C}}}\limits_{\sim},\mathop{{\bf{B}}\hspace{0.0005pt}}\limits_{\sim})$$

$$\begin{array}{c}=\iint p({D}_{1}|\mathop{{\bf{C}}}\limits_{\sim},\mathop{{\bf{B}}\hspace{0.0005pt}}\limits_{\sim},{\mu }_{1},{\sigma }_{1}^{2})p({\mu }_{1},{\sigma }_{1}^{2})d{\mu }_{1}d{\sigma }_{1}^{2}\\ \,\times \iint p({D}_{2}|\mathop{{\bf{C}}}\limits_{\sim},\mathop{{\bf{B}}\hspace{0.0005pt}}\limits_{\sim},{\mu }_{2},{\sigma }_{2}^{2})p({\mu }_{2},{\sigma }_{2}^{2})d{\mu }_{2}d{\sigma }_{2}^{2}\\ \,\times \cdots \\ \,\times \iint p({D}_{I}|\mathop{{\bf{C}}}\limits_{\sim},\mathop{{\bf{B}}\hspace{0.0005pt}}\limits_{\sim},{\mu }_{I},{\sigma }_{I}^{2})p({\mu }_{I},{\sigma }_{I}^{2})d{\mu }_{I}d{\sigma }_{I}^{2}\end{array}$$

$$=\mathop{\prod }\limits_{i=1}^{I}\{\iint N({D}_{i}|{\mu }_{i},{\sigma }_{i}^{2})\times N({\mu }_{i}|{\mu }_{0{C}_{i}^{s}},\frac{{\sigma }_{i}^{2}}{{\kappa }_{{C}_{i}^{s}}})\times IG({\sigma }_{i}^{2}|{\alpha }_{{C}_{i}^{s}},{\beta }_{{C}_{i}^{s}})d{\mu }_{i}d{\sigma }_{i}^{2}\}$$

$$=\mathop{\prod }\limits_{m=1}^{M}\{\begin{array}{c}{(2\pi )}^{\frac{-{L}_{m}}{2}}\times \frac{\sqrt{{\kappa }_{{C}_{m}^{s}}}}{\sqrt{{\kappa }_{{C}_{m}^{s}}+{L}_{m}}}\times \frac{\varGamma ({\alpha }_{{C}_{m}^{s}}+\frac{{L}_{m}}{2})}{\varGamma ({\alpha }_{{C}_{m}^{s}})}\\ \times \frac{{\beta }_{{C}_{m}^{s}}^{{\alpha }_{{C}_{m}^{s}}}}{{({\beta }_{{C}_{m}^{s}}+\frac{{\kappa }_{{C}_{m}^{s}}{\mu }_{0{C}_{m}^{s}}^{2}+\mathop{\sum }\limits_{i={L}_{0}+\mathrm{..}.+{L}_{m-1}+1}^{{L}_{1}+\mathrm{..}.+{L}_{m}}{D}_{i}^{2}-\frac{{({\kappa }_{{C}_{m}^{s}}{\mu }_{0{C}_{m}^{s}}+\mathop{\sum }\limits_{i={L}_{0}+\mathrm{..}.+{L}_{m-1}+1}^{{L}_{1}+\mathrm{..}.+{L}_{m}}{D}_{i})}^{2}}{{\kappa }_{{C}_{m}^{s}}+{L}_{m}}}{2})}^{{\alpha }_{{C}_{m}^{s}}+\frac{{L}_{m}}{2}}}\end{array}\}$$

where *L*_*m*_ is defined as the number of windows in CN region *m*, and *L*_0_ = 0. The settings of hyperparameters ${\mathop{{\boldsymbol{\mu }}}\limits_{\sim}}_{{\bf{0}}}$, $\mathop{{\boldsymbol{\alpha }}\hspace{0.0005pt}}\limits_{\sim}$, $\mathop{{\boldsymbol{\beta }}\hspace{0.0005pt}}\limits_{\sim}$, and $\mathop{{\boldsymbol{\kappa }}\hspace{0.0005pt}}\limits_{\sim}$ are shown in Supplementary Text 1 (Hyperparameters setting).

### Proportional posterior distribution

By multiplying the window boundary, CN state, and depth parts mentioned above, the proportional posterior distributions of $\mathop{{\bf{B}}\hspace{0.0005pt}}\limits_{\sim}$ and $\mathop{{\bf{C}}}\limits_{\sim}$ are obtained.

$$p(\mathop{{\bf{B}}}\limits_{\sim},\mathop{{\bf{C}}}\limits_{\sim}|\mathop{{\bf{D}}}\limits_{\sim})\propto \mathop{\prod }\limits_{m=1}^{M}\{\begin{array}{c}{(2\pi )}^{\frac{-{L}_{m}}{2}}\times \frac{\sqrt{{\kappa }_{{C}_{m}^{s}}}}{\sqrt{{\kappa }_{{C}_{m}^{s}}+{L}_{m}}}\times \frac{\varGamma ({\alpha }_{{C}_{m}^{s}}+\frac{{L}_{m}}{2})}{\varGamma ({\alpha }_{{C}_{m}^{s}})}\\ \times \frac{{\beta }_{{C}_{m}^{s}}^{{\alpha }_{{C}_{m}^{s}}}}{{({\beta }_{{C}_{m}^{s}}+\frac{{\kappa }_{{C}_{m}^{s}}{\mu }_{0{C}_{m}^{s}}^{2}+\mathop{\sum }\limits_{i={L}_{0}+\mathrm{..}.+{L}_{m-1}+1}^{{L}_{1}+\mathrm{..}.+{L}_{m}}{D}_{i}^{2}-\frac{{({\kappa }_{{C}_{m}^{s}}{\mu }_{0{C}_{m}^{s}}+\mathop{\sum }\limits_{i={L}_{0}+\mathrm{..}.+{L}_{m-1}+1}^{{L}_{1}+\mathrm{..}.+{L}_{m}}{D}_{i})}^{2}}{{\kappa }_{{C}_{m}^{s}}+{L}_{m}}}{2})}^{{\alpha }_{{C}_{m}^{s}}+\frac{{L}_{m}}{2}}}\end{array}\}$$

$$\times \{\mathop{\prod }\limits_{k=1}^{K}\frac{\Gamma ({C}_{1k}+{w}_{0k})}{\Gamma ({w}_{0k})}\}\times \mathop{\prod }\limits_{k\text{'}=1}^{K}\{\frac{1}{\Gamma (1+{n}_{k\text{'}})}\mathop{\prod }\limits_{\begin{array}{c}k=1\\ k\ne k\text{'}\end{array}}^{K}\frac{\Gamma ({n}_{k\text{'}k}+\frac{{w}_{0k}}{1-{w}_{0k\text{'}}})}{\Gamma (\frac{{w}_{0k}}{1-{w}_{0k\text{'}}})}\}\times {\lambda }^{M-1}\times {(1-\lambda )}^{I-M}$$

The relationships among the parameters are depicted in Fig. S1.

### Reversible-jump Markov chain Monte Carlo algorithm

Two groups of variables, i.e., CN states $\mathop{{\bf{C}}}\limits_{\sim}$ and window boundaries $\mathop{{\bf{B}}\hspace{0.0005pt}}\limits_{\sim}$, are estimated from the derived posterior distribution $p(\mathop{{\bf{B}}\hspace{0.0005pt}}\limits_{\sim},\mathop{{\bf{C}}}\limits_{\sim}|\mathop{{\bf{D}}}\limits_{\sim})$. In our model, the number of parameters is not fixed, primarily because the values of $\mathop{{\bf{B}}\hspace{0.0005pt}}\limits_{\sim}$ can affect the numbers of CN regions and corresponding states $\mathop{{\bf{C}}}\limits_{\sim}$. A powerful algorithm, i.e., RJMCMC^55^, is adopted for sampling from a specified distribution with a variable number of dimensions. We construct a RJMCMC algorithm with two efficient moves, i.e., “Update copy number states $\mathop{{\bf{C}}}\limits_{\sim}$” and “Update window boundaries $\mathop{{\bf{B}}\hspace{0.0005pt}}\limits_{\sim}$,” for each transition. The details are illustrated below.

To update CN states $\mathop{{\bf{C}}}\limits_{\sim}$, all analyzed regions are updated together via a Gibbs sampler. Conditional on the values of boundaries $\mathop{{\bf{B}}\hspace{0.0005pt}}\limits_{\sim}$ and RDSs $\mathop{{\bf{D}}}\limits_{\sim}$, the probabilities of all possible CN state combinations are calculated. The combination with the maximum probability is selected. The conditional probability is expressed as follows:

$$P(\mathop{{\bf{C}}}\limits_{\sim}|\mathop{{\bf{D}}}\limits_{\sim},\mathop{{\bf{B}}}\limits_{\sim})\propto \mathop{\prod }\limits_{m=1}^{M}\left\{\frac{\sqrt{{\kappa }_{{C}_{m}^{s}}}}{\sqrt{{\kappa }_{{C}_{m}^{s}}+{L}_{m}}}\frac{\varGamma \left({\alpha }_{{C}_{m}^{s}}+\frac{{L}_{m}}{2}\right)}{\varGamma ({\alpha }_{{C}_{m}^{s}})}\frac{{\beta }_{{C}_{m}^{s}}^{{\alpha }_{{C}_{m}^{s}}}}{{\left({\beta }_{{C}_{m}^{s}}+\frac{{\kappa }_{{C}_{m}^{s}}{\mu }_{0{C}_{m}^{s}}^{2}+\mathop{\sum }\limits_{i={L}_{0}+\mathrm{..}.+{L}_{m-1}+1}^{{L}_{1}+\mathrm{..}.+{L}_{m}}{D}_{i}^{2}-\frac{{({\kappa }_{{C}_{m}^{s}}{\mu }_{0{C}_{m}^{s}}+\mathop{\sum }\limits_{i={L}_{0}+\mathrm{..}.+{L}_{m-1}+1}^{{L}_{1}+\mathrm{..}.+{L}_{m}}{D}_{i})}^{2}}{{\kappa }_{{C}_{m}^{s}}+{L}_{m}}}{2}\right)}^{{\alpha }_{{C}_{m}^{s}}+\frac{{L}_{m}}{2}}}\right\}$$

$$\times \left\{\mathop{\prod }\limits_{k=1}^{K}\frac{\Gamma ({C}_{1k}+{w}_{0k})}{\Gamma ({w}_{0k})}\right\}\times \mathop{\prod }\limits_{k\text{'}=1}^{K}\left\{\frac{1}{\Gamma (1+{n}_{k\text{'}})}\mathop{\prod }\limits_{\begin{array}{c}k=1\\ k\ne k\text{'}\end{array}}^{K}\frac{\Gamma \left({n}_{k\text{'}k}+\frac{{w}_{0k}}{1-{w}_{0k\text{'}}}\right)}{\Gamma \left(\frac{{w}_{0k}}{1-{w}_{0k\text{'}}}\right)}\right\}$$

**Figure 3:**
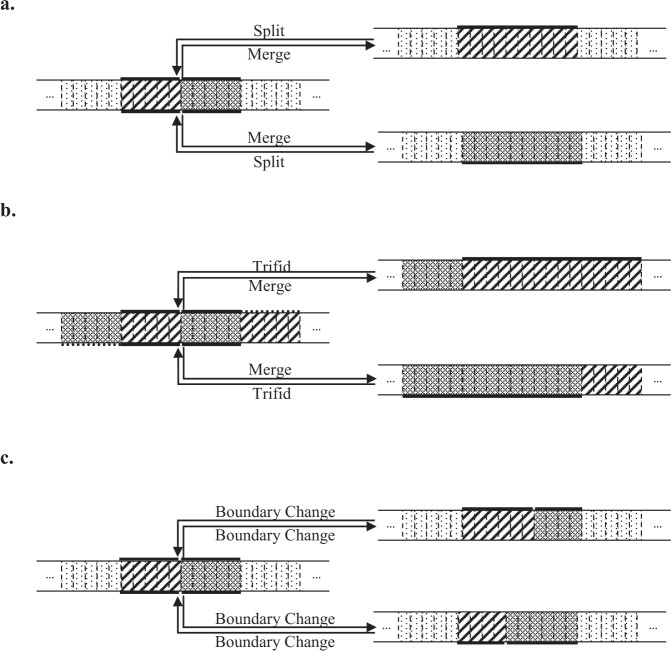
Jumping strategies for updating the copy number boundaries in the RJMCMC algorithm. (**a**) Merge and split, (**b)** merge (double merge) and trifid, and (**c)** boundary change. Each rectangular box indicates a window, and the texture indicates the state of the CN. Continuous windows with the same state are combined into a CN region. For example, in a the slash and argyle CN regions in the left graph are combined into a single region through the merge strategy. One of the original states is assigned to the new region shown in the right graph. Conversely, the slash or argyle region on the right graph is divided into two regions through the split strategy on the left graph.

However, updating window boundaries $\mathop{{\bf{B}}\hspace{0.0005pt}}\limits_{\sim}$ is complex. Because the values of the window boundaries are subject to the dimension of the CN regions and corresponding states, not only the boundaries but also the neighboring CN states are updated in this move. To explore the parameter space efficiently and completely, four novel jumping strategies are adopted: merge, split, trifid, and boundary change. The relationships among the jumping strategies are illustrated in Fig. 3.

In the merge strategy, one window boundary with value 1 (i.e., CN region breakpoint) is randomly changed to a value of 0, and then, the adjacent CN regions sharing the selected boundary are combined. The state of the new CN region is chosen from two states of the original CN regions with equal probability. Assume that the *m* and *m* + 1 regions are merged into a new region with index m*. Then, the candidate status is accepted with the acceptance probability min{1, *A*_*M*1_}. Furthermore, if the state of the newly combined region is accidentally equal to the state of the adjacent region, we automatically merge these regions with the same CN state as a double merge. Two situations require a double merge. First, the *m* and *m* + 1 regions are merged into region m*, and the state of the combined region is selected from *m*. If the state of the *m* + 2 region is equal to that of m*, we merge the m* and *m* + 2 regions into the new region m**. Then, the accepted probability is min{1, *A*_*M*2.1_}. Second, the *m* and *m* + 1 regions are merged to region m*, and the state of the combined region is selected from *m* + 1. If the state of the *m* − 1 region is equal to that of m*, we merge the m* and *m-*1 regions into the new region (*m* − 1)**. Then, the accepted probability is min{1, *A*_*M*2.2_}.

For the reverse strategy named split, one window boundary with a value of 0 (i.e., not a CN region breakpoint) is updated to a value of 1, and then, the CN region is split into two regions. The state of one newly formed region is randomly set to be the same as that of the original region, and the other region is restricted to be unequal to the states of the original and adjacent regions. Assume the selected window boundary belongs to the CN region *m* and that the region *m* is split to m* and m**. Then, the accepted probability is min{1, *A*_*S*_}.

Moreover, the reverse strategy of double merge, which is named trifid (split into three), essentially changes the values of two of the window boundaries with 0 values in one CN region (assuming the *m*^th^ CN region) to 1 values, and then, three CN regions (indexed as m*, m**, and m***) are constructed. The states of the leftmost (m*) and rightmost (m***) regions are assigned to be the same as that of the original region, and the state of the middle region (m**) is randomly selected from the other states with equal probability. The accepted probability is min{1, *A*_*T*_}.

Finally, the breakpoint of the CN region randomly shifts to the left or right window boundary with equal probability without changing the CN states for the boundary change strategy. The accepted probability of the left and right shift are min{1, *A*_*B*−1_} and min{1, *A*_*B*+1_}, respectively. All of the above acceptance probabilities are derived in Supplementary Text 2 (Acceptance probabilities).

For setting the initial values of $\mathop{{\bf{C}}}\limits_{\sim}$ and $\mathop{{\bf{B}}\hspace{0.0005pt}}\limits_{\sim}$ in RJMCMC, a cubic smoothing spline model is fitted to the ordered read-depth signals (RDSs) across the windows. If the predicted RDSs in adjacent windows *i* and *i* + 1 are crossed to the threshold, then the window breakpoint *B*_*i*_ is initially set to 1. The thresholds are approximately defined as the 5^th^ and 95^th^ percentiles of all predicted RDSs. According to the initial breakpoints, we randomly assign the initial state of each region, but the restriction of neighboring regions with different states should be satisfied. The probabilities of selecting four jumping strategies for updating $\mathop{{\bf{B}}\hspace{0.0005pt}}\limits_{\sim}$ are set (as 1/3, 1/6, 1/6 and 1/3).

Additionally, to reduce the unbalanced effect that results from the extreme normal/abnormal state proportion, the whole genome is partitioned into several nonoverlapping sections to estimate the parameters. In our proposed procedure, we run RJMCMC for one genomic section at a time but set the initial values and hyperparameters based on whole genome to ensure that the evidence is sufficient. Advice regarding the section length is provided in the Results section.

### Identification of copy number variations

The samples generated from the posterior distribution through RJMCMC are summarized to identify the CN states of windows via Bayesian testing statistics and Bayes factor (BF)^67^. After burn-in (*t*_*burn*_ = 5,000 iterations, as the default setting), *K* − 1 types of BFs representing the strength for the abnormal states (CN = 1, 3, 4, …, K) against the normal state (CN = 2) in each window are calculated. The BF of window *i* at iteration *t* with abnormal CN state *k* is defined as

$$B{F}_{ti(k)}=\frac{post{r}_{t}({S}_{i}=k)/post{r}_{t}({S}_{i}=2)}{prior({S}_{i}=k)/prior({S}_{i}=2)}$$

where

$$post{r}_{t}({S}_{i}=k)=\frac{{\rm{\#}}\,{\rm{o}}{\rm{f}}\,{\rm{i}}{\rm{t}}{\rm{e}}{\rm{r}}{\rm{a}}{\rm{t}}{\rm{i}}{\rm{o}}{\rm{n}}{\rm{s}}\,{\rm{f}}{\rm{r}}{\rm{o}}{\rm{m}}\,{t}_{burn}\,{\rm{t}}{\rm{o}}\,t\,{\rm{w}}{\rm{i}}{\rm{t}}{\rm{h}}\,{\rm{s}}{\rm{t}}{\rm{a}}{\rm{t}}{\rm{e}}\,k\,{\rm{f}}{\rm{o}}{\rm{r}}\,{\rm{w}}{\rm{i}}{\rm{n}}{\rm{d}}{\rm{o}}{\rm{w}}\,i}{t-{t}_{burn}}\,{\rm{a}}{\rm{n}}{\rm{d}}\,prior({S}_{i}=k)={w}_{0k}$$

BF is derived as

$$B{F}_{ti(k)}=\frac{\#\,{\rm{of}}\,{\rm{iterations}}\,{\rm{from}}\,{t}_{burn}\,{\rm{to}}\,t\,{\rm{with}}\,{\rm{state}}\,k}{\#\,{\rm{of}}\,{\rm{iterations}}\,{\rm{from}}\,{t}_{burn}\,{\rm{to}}\,t\,{\rm{with}}\,{\rm{state}}\,2}\times \frac{{w}_{02}}{{w}_{0k}}$$

*, k* = 1, 3, 4, …, *K*. If the maximum BF in each window is larger than the threshold at the default of 20, decisive evidence is provided that the analytic window has an abnormal CN state $j={\rm{\arg }}\,{\rm{\max }}(B{F}_{ti(1)},B{F}_{ti(3)},\cdots ,B{F}_{ti(K)})$; otherwise, the window is assigned to the normal state. The state of each window is evaluated every 1,000 iterations after burn-in. If all windows remain in the same state over the next 1,000 iterations, then the estimators are stable, and the sampling procedure could be stopped.

Adjacent windows with the same CN state are combined into a CN region. We can then identify the boundaries lying between two CN regions as the CNV breakpoints. However, these observed breakpoints may be just due to a single frequently occurring CNV or due to several CNVs with distinct breakpoints that overlap partially^68^. In fact, read depth methods are poor at localizing breakpoints^69^. Addition information (e.g., partially-mapped reads^70^) and/or computational strategies for merging the genomic regions with a similar copy number^71^ are needed to identify accurate CNV breakpoints. Therefore, current version of CONY does not provide CNV breakpoint prediction.

### Metrics for performance evaluation

The performance of various algorithms is evaluated in terms of the base accuracy and CNV detection rate. In the 1000 Genomes Project analysis, the base accuracy is assessed by three numerical measurements, including the base recall (also called sensitivity), base false positive rate (FPR) and base precision. The base recall is defined as the percentage of basepairs listed as CNVs (i.e., CNV basepairs) in the DGV that are also identified by the algorithm. The base FPR is the percentage of basepairs not listed as CNVs in the DGV that are yet identified as CNVs by the algorithm. The base precision is the percentage of basepairs identified as CNVs by the algorithm that are also listed as CNVs in the DGV. All these metrics evaluate per basepair performance. The CNV detection rate represents the recall for CNV regions, which is the percentage of CNV regions in the DGV that have any position identified as a CNV via the algorithm. The CNV region precision and FPR are not calculated since CONY does not provide exact CNV regions and the DGV is only suitable for defining true positives.

For the simulation study, the base accuracy includes the overall base accuracy, base recall and base FPR. The overall base accuracy is summarized from the correctly identified basepairs. The base recall is defined as the percentage of CNV basepairs that are detected correctly. The base FPR is determined by the percentage of normal basepairs that are classified as copy losses or gains. In addition, the CNV detection rates are calculated for each combination of 2 CNV types (copy loss/gain) versus 10 CNV sizes (1, 2.5, 5, 10, 25, 50, 100, 250, 500 and 1000 kb). If the artificial CNV region is partially or fully identified, then the region is counted. Then, the detection rate is the percentage of detected artificial CNV regions averaged over 100 case samples (for the single-sample analysis) or 100 case-control pairs (for the paired-samples analysis).

## Results

### Application to samples from the 1000 Genomes Project

For NA12156 and NA12878, after the preprocessing steps, approximately 220 megabasepairs (Mb) on chromosome 1 remained for the subsequent analysis. In CONY, approximately 440 sections with 0.5 Mb each were operated in parallel for RJMCMC sampling. The number of possible CN statuses was assigned as 5 (CN 1, 2, 3, 4, and 5) for the single-sample analysis and 3 (copy loss, normal, and copy gain) for the paired-samples analysis. The other parameter settings followed the default settings (see Supplementary Text 1). Some commonly used tools based on read depths (with hundreds of citations through March 2020) were compared. The competing algorithms (CNVnator^28^, FREEC^29^, and rdxplorer^36^ for the single-sample analysis and CNVSeq^35^ and FREEC^29^ for the paired-samples analysis) used the default settings of each tool.

The CNVs identified via each tool were compared with the summarized lists in the Database of Genomic Variants^6^. The searching criteria for DGV were as follows: variant type = CNV, assembly = GRCh37/hg19, cohort name = 1000 Genomes, and the corresponding sample id. CNV regions smaller than 1,000 bp were removed. In summary, 36 CNV regions with 407,253 bp were reported in the DGV for NA12156, and 30 CNV regions with 221,597 bp for NA12878. There were also 36 relative CNV regions with 515,073 bp for NA12156 that was compared with NA12878, and 36 relative CNV regions with 515,073 bp for NA12978 compared with N12156. The numbers of basepairs and CNV regions with CNVs listed in the DGV that were also identified by the algorithms are reported in Table 1. In addition, various metrics for performance evaluation are shown.

**Table 1:** Performance of CNV detection in the experimental data analysis for NA12156 and NA12878.

Sample(s)	Algorithm	CNV bases				CNV regions		Sample(s)	Algorithm	CNV bases				CNV regions	
		bp^a^	Recall	FPR	Precision	Region^b^	Detection rate			bp^a^	Recall	FPR	Precision	Region^b^	Detection rate
**Single-sampleanalysis** (NA12156)	CONY	371,984	91.34%	9.71%	1.68%	33	91.67%	**Single-sample analysis** (NA12878)	CONY	202,607	91.43%	0.51%	1.65%	25	83.33%
	CNVnator	343,308	84.30%	13.07%	1.15%	25	69.44%		CNVnator	168,094	75.86%	0.54%	1.31%	11	36.67%
	FREEC	86,204	21.17%	11.19%	0.34%	2	5.56%		FREEC	124,272	56.08%	0.46%	1.13%	2	6.67%
	rdxplorer	284,865	69.95%	2.28%	5.27%	11	30.56%		rdxplorer	119,034	53.72%	2.17%	0.23%	7	23.33%
	**DGV**	**407,253**				**36**			**DGV**	**221,597**				**30**	
**Paired-samples analysis** (Case:NA12156/ Control:NA12878)	CONY	376,510	73.10%	0.74%	18.55%	23	63.89%	**Paired- samples analysis** (Case:NA12878/ Control:NA12156)	CONY	355,947	69.11%	4.57%	0.32%	25	69.44%
	CNVSeq	163,695	31.78%	15.44%	0.47%	29	80.56%		CNVSeq	175,150	34.00%	0.50%	1.48%	33	91.67%
	FREEC	178,282	34.61%	6.63%	1.18%	3	8.33%		FREEC	230,142	44.68%	1.62%	0.59%	6	16.67%
	**DGV**	**515,073**				**36**			**DGV**	**515,073**				**36**	

^a^The number of CNV basepairs in the DGV that are also identified by the algorithm.

^b^The number of CNV regions in the DGV that have any position identified as a CNV via the algorithm.

In the single-sample analysis for NA12156, more than 90% of the DGV-reported CNV regions were identified by CONY. Notably, of the 407,253 CNV basepairs in the DGV, 371,984 bp (91.34%) was also detected via CONY. CNVnator and rdxplorer identified approximately 80% and 70% of the CNV positions, respectively. FREEC identified only a few validated regions. For basepairs not listed as CNVs in the DGV, rdxplorer identified only 2.28% of them as CNVs while other methods identified about 10%. Disappointingly, all methods performed poorly in precision. Many of the CNVs identified by them were not listed as CNVs in the DGV. Results from NA12878 are generally similar to the findings above.

In the paired-samples analysis for NA12156/NA12878, CONY detected 23 of 36 CNV regions. Although the number of regions was less than that detected by CNVSeq (29 of 36), the proportion of identified CNV positions via CONY (73.10%) was twice that detected by CNVSeq (31.78%). Thus, CNVSeq merely identified a small part of each CNV region. FREEC identified only 3 validated regions, but the proportion of the identified regions (34.61%) was higher than that using CNVSeq. Furthermore, CONY had the lowest FPR and the highest precision among all compared algorithms. CONY’s precision was still low in the paired-samples analysis; it is about 10 times higher than that from the single-sample analysis though. Analyzing paired-samples NA12878/NA12156 leads to results similar to the findings from NA12156/NA12878.

Another two experimental samples (HG00419 and HG01595) with both low- (5.2 to 9.8X) and high- (33.6 to 35.4X) coverage sequencing reads were also analyzed to show consistency of results across samples. These results can be found in Supplementary Table S1. The results from the single-sample and paired-samples analyses in both the low- and high-coverage sequencing data are generally similar to the findings from NA12156 and NA12878.

Overall, CONY detected many more validated CNV regions and positions in both the single-sample and paired-samples analyses than the comparative algorithms in the experimental data analysis. CNV positions identified by CONY but not listed in the DGV were also fewer than those by other algorithms. Among all CNV positions identified by the evaluated algorithms, many of them were not listed as CNVs in the DGV.

### Algorithm performance comparisons in a simulation study

The performance of the proposed procedure, CONY, was also compared with that of published methods for a single-sample analysis (CNVnator, FREEC, and rdxplorer) and paired-samples analysis (CNVSeq and FREEC) on simulation data. The competing algorithms utilized the default settings to identify the CNVs.

**Figure 4:**
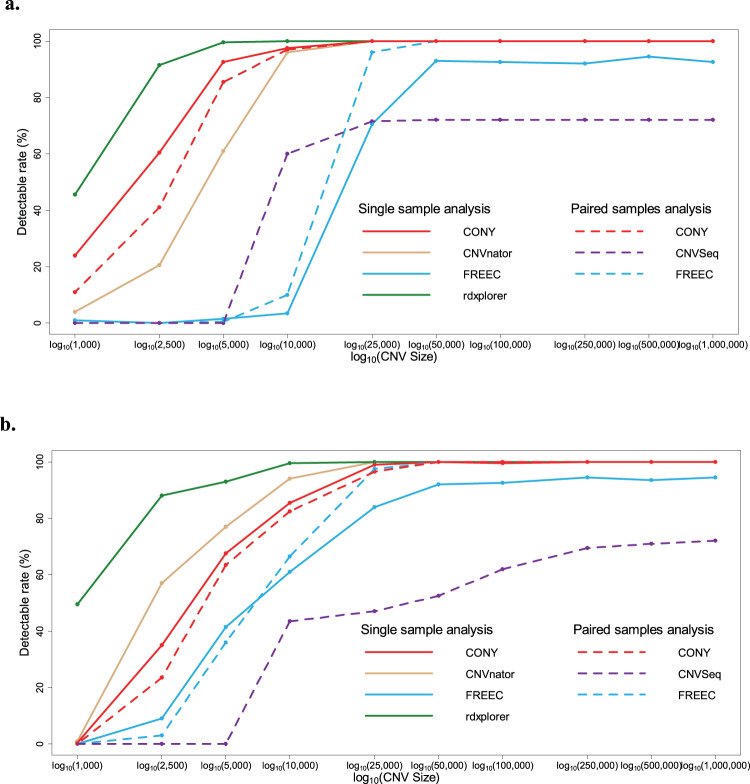
Detection rates of different sizes of CNVs. (**a**) Copy loss results, and (**b)** copy gain results. The solid lines indicate the methods used for the single-sample analysis, and the dashed lines indicate the methods used for the paired-samples analysis.

In the single-sample analysis, CONY performed satisfactorily in terms of overall base accuracy and base recall (Table 2). This comprehensive algorithm also had impressive CNV detection rates, especially for CNV sizes larger than 10 kb (Fig. 4a,b). The testing-based tool rdxplorer revealed great detection rates for all sizes of CNVs. However, the inaccurate breakpoints of the identified CNV regions yielded a low recall and high FPR. CNVnator was too rigorous to detect small CNVs (<10 kb), but its great performance in terms of the detection rates of the midsized and large CNVs contributed to its high overall base accuracy. Notably, CNVnator had high FPRs in detecting the absolute copy loss. FREEC had the worst performance in terms of the CNV detection rates for all sizes among all comparative methods. Overall, the methods had relatively high FPRs in deletion detection compared with duplication detection since copy loss was easier to identify than gain in sequencing platforms^72^. In the paired-samples analysis, CONY was superior to the other methods in terms of CNV detection rates. While FREEC had slightly greater duplication recall than CONY, FREEC was significantly worse at finding small CNVs. CNVseq had a limited ability to detect CNVs.

**Table 2:** Performance comparisons in the simulation study.

(%)	Algorithm	Overall accuracy	Copy loss		Copy gain	
			Recall	FPR	Recall	FPR
**Single-sample analysis**	CONY	99.21	99.44	0.70	97.85	0
	CNVnator	99.09	99.67	0.88	99.11	0.04
	FREEC	98.62	91.77	0.36	92.79	0.02
	rdxplorer	97.12	93.67	0.59	82.18	0.18
**Paired-samples analysis**	CONY	99.86	99.50	0.02	98.82	0.01
	CNVSeq	94.65	71.10	0.01	47.35	0.01
	FREEC	99.72	98.49	0.06	98.90	0.07

In summary, CONY can detect both absolute and relative CNVs in single- and paired-samples analyses. CNVs with moderate to large sizes (>10 kb) can almost completely be detected by CONY. However, detecting small CNVs using a read-depth-based algorithm, including CONY, is challenging. The detection rates of small CNVs can be greatly improved by increasing the read coverage, which is demonstrated in the following results. Due to the poor power for detecting small CNVs for low-read-coverage data (e.g., 2.2X in our simulation), we suggest focusing on detecting CNVs with sizes >1,000 bp (as per the usual definition) to reduce potential false positives.

All simulations were run via the supercomputer Advanced Large-scale Parallel Supercluster (ALPS) at the National Center for High-performance Computing, National Applied Research Laboratories, Taiwan, with an AMD Opteron 6174 2.2 GHz × 4 CPU, a DDR3 ECC 128 GB of memory, and 512 nodes. In the RJMCMC procedure, one chromosome was divided into several nonoverlapping sections of equal size 0.5 Mb, and the operations were performed in parallel. The running time corresponded to the components of the CN in each analytic section. If only one CN state was included in the section, then the computing time was less than 1 minute. For a section with complex CN components, in our experience, the greatest length of time until RJMCMC became stable was less than 10 minutes. The running time for the other competing approaches with complex CN components are shown below: rdxplorer (~4 minutes), CNVnator (~15 minutes), FREEC (10 to 20 minutes), and CNVSeq (2 to 3 hours).

### Analytic section length decision

To address the unbalanced structure of normal/variant regions in the genome, the whole genome can be partitioned into several nonoverlapping sections to estimate the parameters. The optimal section length for RJMCMC was derived via simulation. The samples generated for the algorithm comparisons in the above section were used. Six analytic lengths were adopted, including 60, 10, 5, 1, 0.5, and 0.1 Mb per section. Both the CNV detection rate and the base accuracy were used to select the proper section lengths.

Supplementary Fig. S2 presents the CNV detection rates using various CNV sizes and section lengths. As shown in the figure, the detection rates of CNVs of various sizes were enhanced by reducing the analytic section lengths. However, enhancing the detection rate appreciably for small CNVs (<10 kb) was challenging, even after shrinking the section lengths. CNVs larger than 10 kb were considered to select an optimal section length. If the minimum requirement of the detection rate was set as 80%, then the section length should be shorter than 0.5 Mb. If a more severe detection rate was set, then a shorter section size was needed. In terms of CNV detection ability, the optimal section size was considered to be less than 0.5 Mb.

For the base accuracy, the results are shown in Supplementary Table S2. The recall was improved by shortening the section lengths. However, the FPRs dramatically increased when the sections were too small to provide sufficient evidence. In terms of the overall base accuracy, approximately 0.5–1 Mb (for single-sample analysis) and 0.1–0.5 Mb (for paired-samples analysis) were the proper section lengths for achieving peak accuracy. Based on the two performance measurements mentioned above, the recommendations for the section length were simplified to 0.5 Mb, which was also adopted in our experimental data analysis and simulation studies.

### Window read-depth estimation

In this study, an alternative method was adopted for window read-depth estimation to enhance the completeness of the genetic information. Traditionally, the middle or start position of a read is located in a specific window, and the read is counted for the depth of this window. However, this strategy might underestimate the contribution of reads that span many windows. In our procedure, a summation approach was used. The read depths of each base were generated using the piling procedure in SAMtools, and then, the base depths in the specific window were summed as the window read depth. Supplementary Table S3 provides evidence that the summation method can improve both the CNV detection rate and the overall accuracy compared with the traditional representative-position method in single-sample analyses, especially for low coverage sequencing.

### Read coverage

Because the NGS platform is still more expensive than other available technologies, researchers might process several samples in a single experimental run, which can result in low coverage. The depths based on sparse read coverage may lead to insufficient evidence for CNV identification. To evaluate the coverage effect, we followed the simulation settings mentioned above and generated 100 cases that were sequenced with a high coverage (22×). The CNV detection rates and base accuracies in the single-sample analysis are listed in Supplementary Table S3. Obviously, a great improvement was achieved in terms of CNV detection capability with high-coverage sequencing, especially for the detection of small variants. The impressive detection rates and outstanding recalls were attributed to the sufficient data information, but the false discoveries are expected to be accompanied by additional variations. Notably, no obvious differences were observed in the overall base accuracies between the low- (99.21%) and high–coverage (98.74%) data by CONY.

Two experimental samples (HG00419 and HG01595) from the 1000 Genomes Project, which were sequenced with both low (5.2 to 9.8X) and high (33.6 to 35.4X) coverages, were also analyzed to evaluate the coverage effect (Supplementary Table S1). High-coverage sequencing generally achieved better base accuracy and CNV detection rates in both single-sample and paired-samples analyses than low-coverage sequencing did for all tested algorithms. The base recall from CONY in the single-sample analysis is an exception, where high-coverage sequencing did not do better than low-coverage sequencing.

## Discussion

Based on a comprehensive Bayesian hierarchical model and an efficient RJMCMC inference algorithm, the procedure proposed in this article was proven to be robust and precise for CNV detection. This functional tool can be applied for different purposes, including the detection of absolute and relative CNVs under single-sample and paired-samples designs. Samples from the 1000 Genomes Project were analyzed. CONY detected more CNVs and positions validated by the DGV database than the compared algorithms. In the simulation studies, the estimation methods performed well in terms of the overall base accuracy, recall and FPR for both single-sample and paired-samples analyses. Additionally, the CNV detection rates were effectively improved by selecting the proper analytic section length in the RJMCMC method and by adopting summation window read-depth estimation. The detection rates for small CNVs were still restricted even with suitable section lengths and depth estimation. In addition, we showed that the detection of small CNVs can be greatly improved by increasing the read coverage.

Although whole genome sequencing (WGS) is a comprehensive platform for exploring potential variants, target exome sequencing (TES) is an efficient choice because human exons constitute approximately 1% of the total genome^73^ but over 85% of genomic disease-causing regions^74^. Exome sequencing provides effective information with high coverage on a limited budget. Read generation with WGS and TES follows distinct procedures due to the concentrations of DNA, the environments of hybridization and the methods of sequencing. Because of these experimental differences, the algorithms used to detect CNVs from WGS^28–30,35,36^ and TES^29,31–34,75^ are distinct, with alternative preprocessing, bias corrections and model assumptions.

WGS can detect more CNVs and precise breakpoints due to the complete genome scanning. WGS-based methods consider the continuity of the genomic space, and the CNVs are estimated from the read depths across the genome with few significant bias corrections, such as for potential PCR duplicates and GC content. In contrast, the prediction of exact CNV breakpoints and small CNVs by segmentation algorithms in interrupted target exome sequences is challenging. In addition, exon-specific biases, such as exon sizes and batch and background effects, need to be corrected via multiple sample comparisons and/or additional adjustment steps. Therefore, the existing methods of WGS and TES seldom have commonalities. Modifying our approach for both WGS and TES under a common model framework will be a challenge for future research.

## Supplementary information

Supplementary Information.

## Data Availability

The datasets used and analyzed in this study are available from 1000 Genomes Project (http://www.1000genomes.org). R code that implements the proposed procedure is available at https://github.com/weiyuchung/CONY, with direct links for downloading available at https://github.com/weiyuchung/CONY/archive/master.zip.

## References

[1] Freeman JL (2006). Copy number variation: new insights in genome diversity. Genome research.

[2] Redon R (2006). Global variation in copy number in the human genome. nature.

[3] Girirajan S, Campbell CD, Eichler EE (2011). Human copy number variation and complex genetic disease. Annual review of genetics.

[4] Alkan C, Coe BP, Eichler EE (2011). Genome structural variation discovery and genotyping. Nature Reviews Genetics.

[5] MacDonald JR, Ziman R, Yuen RK, Feuk L, Scherer SW (2013). The Database of Genomic Variants: a curated collection of structural variation in the human genome. Nucleic acids research.

[6] Database of Genomic Variants, http://dgv.tcag.ca/dgv/app/home (2013).

[7] Sebat J (2004). Large-scale copy number polymorphism in the human genome. Science.

[8] Leffler EM (2017). Resistance to malaria through structural variation of red blood cell invasion receptors. Science.

[9] Hollox EJ (2008). Psoriasis is associated with increased β-defensin genomic copy number. Nature genetics.

[10] Stuart PE (2012). Association of β-defensin copy number and psoriasis in three cohorts of European origin. Journal of Investigative Dermatology.

[11] Stefansson H (2014). CNVs conferring risk of autism or schizophrenia affect cognition in controls. Nature.

[12] Heinzen EL (2010). Genome-wide scan of copy number variation in late-onset Alzheimer’s disease. Journal of Alzheimer’s Disease.

[13] Kirov G (2014). The penetrance of copy number variations for schizophrenia and developmental delay. Biological psychiatry.

[14] Cooper GM (2011). A copy number variation morbidity map of developmental delay. Nature genetics.

[15] Chan KA (2013). Cancer genome scanning in plasma: detection of tumor-associated copy number aberrations, single-nucleotide variants, and tumoral heterogeneity by massively parallel sequencing. Clinical chemistry.

[16] Fridlyand J (2006). Breast tumor copy number aberration phenotypes and genomic instability. BMC cancer.

[17] Pan X (2019). Identification of the copy number variant biomarkers for breast cancer subtypes. Molecular Genetics and Genomics.

[18] Salido M (2011). Increased ALK gene copy number and amplification are frequent in non-small cell lung cancer. Journal of thoracic oncology.

[19] Ocak S (2010). DNA copy number aberrations in small-cell lung cancer reveal activation of the focal adhesion pathway. Oncogene.

[20] Xie T (2012). A comprehensive characterization of genome-wide copy number aberrations in colorectal cancer reveals novel oncogenes and patterns of alterations. PloS one.

[21] Diep CB (2006). The order of genetic events associated with colorectal cancer progression inferred from meta‐analysis of copy number changes. Genes, Chromosomes and Cancer.

[22] Lai WR, Johnson MD, Kucherlapati R, Park PJ (2005). Comparative analysis of algorithms for identifying amplifications and deletions in array CGH data. Bioinformatics.

[23] Van de Wiel MA, Picard F, Van Wieringen WN, Ylstra B (2011). Preprocessing and downstream analysis of microarray DNA copy number profiles. Briefings in bioinformatics.

[24] Dellinger AE (2010). Comparative analyses of seven algorithms for copy number variant identification from single nucleotide polymorphism arrays. Nucleic acids research.

[25] Winchester L, Yau C, Ragoussis J (2009). Comparing CNV detection methods for SNP arrays. Briefings in functional genomics & proteomics.

[26] Teo SM, Pawitan Y, Ku CS, Chia KS, Salim A (2012). Statistical challenges associated with detecting copy number variations with next-generation sequencing. Bioinformatics.

[27] Xi R, Kim T-M, Park PJ (2010). Detecting structural variations in the human genome using next generation sequencing. Briefings in functional genomics.

[28] Abyzov A, Urban AE, Snyder M, Gerstein M (2011). CNVnator: an approach to discover, genotype, and characterize typical and atypical CNVs from family and population genome sequencing. Genome research.

[29] Boeva V (2012). Control-FREEC: a tool for assessing copy number and allelic content using next-generation sequencing data. Bioinformatics.

[30] Chiang DY (2009). High-resolution mapping of copy-number alterations with massively parallel sequencing. Nature methods.

[31] Deng X (2011). SeqGene: a comprehensive software solution for mining exome-and transcriptome-sequencing data. BMC bioinformatics.

[32] Koboldt DC (2012). VarScan 2: somatic mutation and copy number alteration discovery in cancer by exome sequencing. Genome research.

[33] Love MI (2011). Modeling read counts for CNV detection in exome sequencing data. Statistical Applications in Genetics and Molecular Biology.

[34] Plagnol V (2012). A robust model for read count data in exome sequencing experiments and implications for copy number variant calling. Bioinformatics.

[35] Xie C, Tammi MT (2009). CNV-seq, a new method to detect copy number variation using high-throughput sequencing. BMC bioinformatics.

[36] Yoon S, Xuan Z, Makarov V, Ye K, Sebat J (2009). Sensitive and accurate detection of copy number variants using read depth of coverage. Genome research.

[37] Chen K (2009). BreakDancer: an algorithm for high-resolution mapping of genomic structural variation. Nature methods.

[38] Hormozdiari F (2010). Next-generation VariationHunter: combinatorial algorithms for transposon insertion discovery. Bioinformatics.

[39] Hormozdiari F, Hajirasouliha I, McPherson A, Eichler EE, Sahinalp SC (2011). Simultaneous structural variation discovery among multiple paired-end sequenced genomes. Genome research.

[40] Korbel JO (2009). PEMer: a computational framework with simulation-based error models for inferring genomic structural variants from massive paired-end sequencing data. Genome Biol.

[41] Zhang ZD (2011). Identification of genomic indels and structural variations using split reads. BMC genomics.

[42] Ye K, Schulz MH, Long Q, Apweiler R, Ning Z (2009). Pindel: a pattern growth approach to detect break points of large deletions and medium sized insertions from paired-end short reads. Bioinformatics.

[43] Abel HJ (2010). SLOPE: a quick and accurate method for locating non-SNP structural variation from targeted next-generation sequence data. Bioinformatics.

[44] Iqbal Z, Caccamo M, Turner I, Flicek P, McVean G (2012). De novo assembly and genotyping of variants using colored de Bruijn graphs. Nature genetics.

[45] Nijkamp JF (2012). De novo detection of copy number variation by co-assembly. Bioinformatics.

[46] Luo R (2012). SOAPdenovo2: an empirically improved memory-efficient short-read de novo assembler. Gigascience.

[47] Medvedev P, Fiume M, Dzamba M, Smith T, Brudno M (2010). Detecting copy number variation with mated short reads. Genome research.

[48] Hajirasouliha I (2010). Detection and characterization of novel sequence insertions using paired-end next-generation sequencing. Bioinformatics.

[49] Handsaker RE, Korn JM, Nemesh J, McCarroll SA (2011). Discovery and genotyping of genome structural polymorphism by sequencing on a population scale. Nature genetics.

[50] Quinlan AR (2010). Genome-wide mapping and assembly of structural variant breakpoints in the mouse genome. Genome research.

[51] Zeitouni B (2010). SVDetect: a tool to identify genomic structural variations from paired-end and mate-pair sequencing data. Bioinformatics.

[52] Zhao M, Wang Q, Wang Q, Jia P, Zhao Z (2013). Computational tools for copy number variation (CNV) detection using next-generation sequencing data: features and perspectives. BMC Bioinformatics.

[53] González JR (2009). Accounting for uncertainty when assessing association between copy number and disease: a latent class model. BMC bioinformatics.

[54] Glessner, J. T., Li, J. & Hakonarson, H. ParseCNV integrative copy number variation association software with quality tracking. *Nucleic acids research*, gks1346 (2013). 10.1093/nar/gks1346 PMC3597648 23293001

[55] Green PJ (1995). Reversible jump Markov chain Monte Carlo computation and Bayesian model determination. Biometrika.

[56] Consortium GP (2010). A map of human genome variation from population-scale sequencing. Nature.

[57] Li H (2009). The sequence alignment/map format and SAMtools. Bioinformatics.

[58] Li H, Durbin R (2009). Fast and accurate short read alignment with Burrows–Wheeler transform. bioinformatics.

[59] Langmead B, Salzberg SL (2012). Fast gapped-read alignment with Bowtie 2. Nature methods.

[60] Ewing B, Green P (1998). Base-calling of automated sequencer traces using phred. II. Error probabilities. Genome research.

[61] Li H, Ruan J, Durbin R (2008). Mapping short DNA sequencing reads and calling variants using mapping quality scores. Genome research.

[62] Salmi, A. *et al*. CNV-LDC: An Optimized CNV Detection Method for Low Depth of Coverage Data. *Bioinformatics*, 37–42 (2017).

[63] Dohm JC, Lottaz C, Borodina T, Himmelbauer H (2008). Substantial biases in ultra-short read data sets from high-throughput DNA sequencing. Nucleic acids research.

[64] Medvedev P, Stanciu M, Brudno M (2009). Computational methods for discovering structural variation with next-generation sequencing. Nature methods.

[65] Gusnanto A, Wood HM, Pawitan Y, Rabbitts P, Berri S (2012). Correcting for cancer genome size and tumour cell content enables better estimation of copy number alterations from next-generation sequence data. Bioinformatics.

[66] Ivakhno S (2010). CNAseg—a novel framework for identification of copy number changes in cancer from second-generation sequencing data. Bioinformatics.

[67] Kass RE, Raftery AE (1995). Bayes factors. Journal of the american statistical association.

[68] Korbel JO (2007). Systematic prediction and validation of breakpoints associated with copy-number variants in the human genome. Proceedings of the National Academy of Sciences.

[69] Medvedev P, Stanciu M, Brudno M (2009). Computational methods for discovering structural variation with next-generation sequencing. Nature methods.

[70] Nord AS, Lee M, King M-C, Walsh T (2011). Accurate and exact CNV identification from targeted high-throughput sequence data. BMC genomics.

[71] Dona MS, Prendergast LA, Mathivanan S, Keerthikumar S, Salim A (2017). Powerful differential expression analysis incorporating network topology for next-generation sequencing data. Bioinformatics.

[72] Xi R (2011). Copy number variation detection in whole-genome sequencing data using the Bayesian information criterion. Proceedings of the National Academy of Sciences.

[73] Ng SB (2009). Targeted capture and massively parallel sequencing of 12 human exomes. Nature.

[74] Choi M (2009). Genetic diagnosis by whole exome capture and massively parallel DNA sequencing. Proceedings of the National Academy of Sciences.

[75] Sathirapongsasuti JF (2011). Exome sequencing-based copy-number variation and loss of heterozygosity detection: ExomeCNV. Bioinformatics.

