# Supplementary material for: CONY: A Bayesian procedure for detecting copy number variations from sequencing read depths
Source: Sci Rep. 2020 Jun 26;10:10493. doi: 10.1038/s41598-020-64353-1 (PMC7319969; doi:10.1038/s41598-020-64353-1)
Supplement: Supplementary file 1 — Supplementary Information. [file 41598_2020_64353_MOESM1_ESM.pdf]

Supplementary Information

CONY: A Bayesian procedure for detecting copy number variations from sequencing read depths

Yu-Chung Wei[1] and Guan-Hua Huang[2,*]

[1]**Yu-Chung Wei**

Graduate Institute of Statistics and Information Science, National Changhua University of Education

No.1 Jinde Road, Changhua City, Changhua County 50007, TAIWAN.

E-mail: weiyuchung@cc.ncue.edu.tw

[2]**Guan-Hua Huang** (*Corresponding Author)

Institute of Statistics, National Chiao Tung University

1001 University Road, Hsinchu 30010, TAIWAN.

E-mail: ghuang@stat.nctu.edu.tw

Supplementary Text 1. Hyperparameter settings

Here, the default settings of the hyperparameters, including λ (for the window boundary part), $\underset{\sim}{\mathbf{W}}_{\mathbf{0}}$ (for the copy number state part), and $\underset{\sim}{\boldsymbol{\mu}}_{\mathbf{0}}$, $\underset{\sim}{\boldsymbol{\alpha}}$, $\underset{\sim}{\boldsymbol{\beta}}$, and $\underset{\sim}{\boldsymbol{\kappa}}$ (for the depth part), are provided.

First, success probability λ is set as a non-informative prior at 0.5 in the window boundary part. The other specified number was available if additional information was provided. Second, the hyperparameters of the CN state and depth parts ($\underset{\sim}{\boldsymbol{\mu}}_{\mathbf{0}}, \underset{\sim}{\boldsymbol{\alpha}}, \underset{\sim}{\boldsymbol{\beta}}, \underset{\sim}{\boldsymbol{\kappa}}, \underset{\sim}{\mathbf{W}}_{\mathbf{0}}$) are considered. The prior means ($\underset{\sim}{\boldsymbol{\mu}}_{\mathbf{0}} = \left[\mu_{01}, \mu_{02}, ..., \mu_{0K}\right]$) and corresponding variances ($\boldsymbol{\sigma}_{\mathbf{0}}^{\mathbf{2}} = \left[\sigma_{01}^2, \sigma_{02}^2, ..., \sigma_{0K}^2\right]$) of RDSs in each CN state are determined via a comprehensive rule. We consider a single-sample analysis as an example. For the normal state (CN: k=2), the mean (μ_{02}) is estimated by the sample median ($\hat{\eta}_{50\%}$), which is robust against potential outliers from extreme CNVs. The identical proportions of copy losses and gains are assumed to be a total of 10%, which is approximately the same as that in the human genome (i.e., the CN state of RDSs between $\hat{\eta}_{5\%}$ and $\hat{\eta}_{95\%}$ is deemed as the potential normal state). Based on the assumption that the RDSs in a specific CN state are normally distributed, the standard deviation of RDSs in the normal state (σ_{02}) is estimated by $\left(\hat{\eta}_{95\%} - \hat{\eta}_{50\%}\right)/\Phi^{-1}\left(95\%\right)$, where $\Phi(.)$ is the cumulative density probability of the standard normal distribution. In summary, the mean and variance estimators of RDSs in the normal CN state are denoted as $\hat{\mu}_{02} = \hat{\eta}_{50\%}$ and $\hat{\sigma}_{02}^2 = \left(\hat{\eta}_{95\%} - \hat{\eta}_{50\%}\right)^2 / \left(\Phi^{-1}\left(95\%\right)\right)^2$.

Moreover, the mean (μ_{0k}) and variance (σ_{0k}^2) of RDSs in the abnormal CN states ($k \neq 2$) are set. The main concept of the proposed model is the window read counts/ratios (exponential RDSs) are proportional to the copy number. Then, the exponential RDSs in a specific CN state

k follow a log-normal distribution with a mean ($\mu_{\exp,0k} = \mu_{\exp,02} \times k/2$) and variance ($\sigma^2_{\exp,0k} = \sigma^2_{\exp,02} \times k/2$). According to the relationship between the normal and log-normal distributions, the general form of the mean and variance of the RDSs can be described by

$$\mu_{0k} = \log\left(\frac{\mu^2_{\exp,0k}}{\sqrt{\sigma^2_{\exp,0k} + \mu^2_{\exp,0k}}}\right) = \frac{k}{2} \times \log\left(\frac{\mu^2_{\exp,02}}{\sqrt{\frac{2}{k}\sigma^2_{\exp,02} + \mu^2_{\exp,02}}}\right),$$

$$\sigma^2{}_{0k} = \log\left(1 + \frac{\sigma^2_{\exp,0k}}{\mu^2_{\exp,0k}}\right) = \log\left(1 + \frac{2}{k} \times \frac{\sigma^2_{\exp,02}}{\mu^2_{\exp,02}}\right),$$

or

$$\mu_{\exp,0k} = \frac{k}{2} \times \mu_{\exp,02} = \frac{k}{2} \times \exp\left(\mu_{02} + \frac{\sigma^2_{02}}{2}\right),\ \sigma^2_{\exp,0k} = \frac{k}{2} \times \sigma^2_{\exp,02} = \frac{k}{2} \times \left(e^{\sigma^2_{02}} - 1\right) e^{2\mu_{02} + \sigma^2_{02}}$$

The estimators are shown as follows:

$$\hat{\mu}_{0k} = \frac{k}{2} \times \log\left(\frac{\left(\hat{\eta}_{50\%}\right)^2}{\sqrt{\frac{2}{k} \times \left(\frac{\hat{\eta}_{95\%} - \hat{\eta}_{50\%}}{\Phi^{-1}(95\%)}\right)^2 + \left(\hat{\eta}_{50\%}\right)^2}}\right) \text{ and } \hat{\sigma}^2_{0k} = \log\left(1 + \frac{2}{k} \times \frac{\left(\frac{\hat{\eta}_{95\%} - \hat{\eta}_{50\%}}{\Phi^{-1}(95\%)}\right)^2}{\left(\hat{\eta}_{50\%}\right)^2}\right) \text{ for } k$$

from 1 to K. For the paired-samples analysis, we use the same rule to calculate the prior mean and variance with copy losses $k = 1$ and gains $k = 3$.

Next, the prior weights of CN states ($\underset{\sim}{\mathbf{w}}_{\mathbf{0}} = [w_{01}, w_{02}, \ldots, w_{0K}]$) are specified. Window *i* is assigned to CN state *k* as the initial state if $\underset{\sim}{D}_i$ is much closer to μ_{0k} than the other CN state means. Then, the proportion of windows belonging to state *k* are summarized to estimate the initial weight w_{0k}. Furthermore, κ_k is represented as the number of windows belonging to CN state *k* and estimated by the estimator of w_{0k} by multiplying the total number of windows

I. The shape parameter α_k is fixed at 5 for all CN states, and the scale parameter is calculated as $\beta_k = (\alpha_k - 1) \times \sigma_{0k}^2 \times \kappa_k / (\kappa_k + 1)$ via a normal-inverse-gamma distribution transformation.

Supplementary Text 2. Acceptance probabilities

To balance the parameter status for the boundary-updating move, the reverse move of each strategy is essential. The four strategies mentioned above are grouped into three types of jumping pairs, including merge/split, double merge/trifid, and boundary changes to the left and right. Each pair is illustrated in Figure 3, and their accepted probabilities are derived as follows.

In the accepted probabilities, $\underset{\sim}{\mathbf{B}}_{(Before)}$ and $\underset{\sim}{\mathbf{B}}_{(After)}$ are the window boundary vectors before and after the update, respectively. $p\left(.\middle|\underset{\sim}{\mathbf{D}}\right)$ is the conditional likelihood, and $q(.)$ is the transition density function between the original and proposed states. $\underset{\sim}{\mathbf{u}}$ (or $\underset{\sim}{\mathbf{u}}_1$, $\underset{\sim}{\mathbf{u}}_2$) is an additional vector used to balance the parameter dimension with the density function $p\left(\underset{\sim}{\mathbf{u}}\right)$ (or $p\left(\underset{\sim}{\mathbf{u}}_1, \underset{\sim}{\mathbf{u}}_2\right)$). The absolute value term is the Jacobian from the parameter change.

First, merge and split are opposite strategies with accepted probabilities $\min\{1, A_{M1}\}$ and $\min\{1, A_S\}$, respectively. In the merge strategy, we randomly select a window boundary with values from 1 to 0. Assume that the breakpoints of regions *m* and *m*+1 are chosen; then, these adjacent regions are merged into a new region, designated as *m**. The CN state of the updated region is randomly chosen from the original states with equal probabilities. Based on this rule, the transition density function $q(.)$ is derived. Moreover, the variable $\underset{\sim}{\mathbf{u}}$ should be fixed when the state of region $m*$ is determined, and its probability is set to 1. In addition, the Jacobian of the transformation equals 1.

For the reverse move split, we randomly select a window boundary that belongs to region *m* to change its value to 1. Then, the region is split into *m** and *m***. One of the two updated regions is randomly selected with an identical probability, and the state of the selected region is assigned to equal that of the original one. The state of the other region is restricted to be unequal to the original and adjacent states. According to the transition rule, the density function

$q(.)$ is derived. Moreover, the variable $\underset{\sim}{\mathbf{u}}$ should also be fixed, and its probability is set to 1. In addition, the Jacobian of the transformation is equal to 1.

Then, A_{M1} and A_S are calculated as follows:

$$A_{M1} = \frac{p\left(\underset{\sim}{\mathbf{B}}_{(After)}, \underset{\sim}{\mathbf{C}}_{\mathbf{1}},\dots,\underset{\sim}{\mathbf{C}}_{\mathbf{m-1}},\underset{\sim}{\mathbf{C}}_{\mathbf{m*}},\underset{\sim}{\mathbf{C}}_{\mathbf{m+2}},\dots,\underset{\sim}{\mathbf{C}}_{\mathbf{M}} \middle| \underset{\sim}{\mathbf{D}}\right)}{p\left(\underset{\sim}{\mathbf{B}}_{(Before)}, \underset{\sim}{\mathbf{C}}_{\mathbf{1}},\dots,\underset{\sim}{\mathbf{C}}_{\mathbf{m-1}},\underset{\sim}{\mathbf{C}}_{\mathbf{m}},\underset{\sim}{\mathbf{C}}_{\mathbf{m+1}},\dots,\underset{\sim}{\mathbf{C}}_{\mathbf{M}} \middle| \underset{\sim}{\mathbf{D}}\right)}$$

$$\times \frac{q\left(\underset{\sim}{\mathbf{B}}_{(Before)}, \underset{\sim}{\mathbf{C}}_{\mathbf{1}},\dots,\underset{\sim}{\mathbf{C}}_{\mathbf{M}} \middle| \underset{\sim}{\mathbf{B}}_{(After)}, \underset{\sim}{\mathbf{C}}_{\mathbf{1}},\dots,\underset{\sim}{\mathbf{C}}_{\mathbf{m-1}},\underset{\sim}{\mathbf{C}}_{\mathbf{m*}},\underset{\sim}{\mathbf{C}}_{\mathbf{m+2}},\dots,\underset{\sim}{\mathbf{C}}_{\mathbf{M}}\right) \times p\left(\underset{\sim}{\mathbf{u}}\right)}{q\left(\underset{\sim}{\mathbf{B}}_{(After)}, \underset{\sim}{\mathbf{C}}_{\mathbf{1}},\dots,\underset{\sim}{\mathbf{C}}_{\mathbf{m-1}},\underset{\sim}{\mathbf{C}}_{\mathbf{m*}},\underset{\sim}{\mathbf{C}}_{\mathbf{m+2}},\dots,\underset{\sim}{\mathbf{C}}_{\mathbf{M}} \middle| \underset{\sim}{\mathbf{B}}_{(Before)}, \underset{\sim}{\mathbf{C}}_{\mathbf{1}},\dots,\underset{\sim}{\mathbf{C}}_{\mathbf{M}}\right)}$$

$$\times \left| \frac{\partial\left(\underset{\sim}{\mathbf{B}}_{(After)}, \underset{\sim}{\mathbf{C}}_{\mathbf{1}},\dots,\underset{\sim}{\mathbf{C}}_{\mathbf{m-1}},\underset{\sim}{\mathbf{C}}_{\mathbf{m*}},\underset{\sim}{\mathbf{C}}_{\mathbf{m+2}},\dots,\underset{\sim}{\mathbf{C}}_{\mathbf{M}},\underset{\sim}{\mathbf{u}}\right)}{\partial\left(\underset{\sim}{\mathbf{B}}_{(Before)}, \underset{\sim}{\mathbf{C}}_{\mathbf{1}},\dots,\underset{\sim}{\mathbf{C}}_{\mathbf{M}}\right)} \right|$$

$$= \frac{\frac{\sqrt{\kappa_{C^s_{m*}}}}{\sqrt{\kappa_{C^s_{m*}} + L_{m*}}} \frac{\Gamma\left(\alpha_{C^s_{m*}} + \frac{L_{m*}}{2}\right)}{\Gamma\left(\alpha_{C^s_{m*}}\right)} \frac{\beta_{C^s_{m*}}^{\alpha_{C^s_{m*}}}}{\Psi_{m*}}}{\left\{ \frac{\sqrt{\kappa_{C^s_m}}}{\sqrt{\kappa_{C^s_m} + L_m}} \frac{\Gamma\left(\alpha_{C^s_m} + \frac{L_m}{2}\right)}{\Gamma\left(\alpha_{C^s_m}\right)} \frac{\beta_{C^s_m}^{\alpha_{C^s_m}}}{\Psi_m} \times \frac{\sqrt{\kappa_{C^s_{m+1}}}}{\sqrt{\kappa_{C^s_{m+1}} + L_{m+1}}} \frac{\Gamma\left(\alpha_{C^s_{m+1}} + \frac{L_{m+1}}{2}\right)}{\Gamma\left(\alpha_{C^s_{m+1}}\right)} \frac{\beta_{C^s_{m+1}}^{\alpha_{C^s_{m+1}}}}{\Psi_{m+1}} \right\}}$$

$$\times \frac{\left\{ \prod_{k=1}^{K} \frac{\Gamma\left(C_{1k(After)} + w_{0k}\right)}{\Gamma\left(w_{0k}\right)} \right\} \times \prod_{k'=1}^{K} \left\{ \frac{1}{\Gamma\left(1 + n_{k'(After)}\right)} \prod_{\substack{k=1 \\ k \neq k'}}^{K} \frac{\Gamma\left(n_{k'k(After)} + \frac{w_{0k}}{1 - w_{0k'}}\right)}{\Gamma\left(\frac{w_{0k}}{1 - w_{0k'}}\right)} \right\}}{\left\{ \prod_{k=1}^{K} \frac{\Gamma\left(C_{1k(Before)} + w_{0k}\right)}{\Gamma\left(w_{0k}\right)} \right\} \times \prod_{k'=1}^{K} \left\{ \frac{1}{\Gamma\left(1 + n_{k'(Before)}\right)} \prod_{\substack{k=1 \\ k \neq k'}}^{K} \frac{\Gamma\left(n_{k'k(Before)} + \frac{w_{0k}}{1 - w_{0k'}}\right)}{\Gamma\left(\frac{w_{0k}}{1 - w_{0k'}}\right)} \right\}}$$

$$\times \frac{1-\lambda}{\lambda} \times \left(\begin{array}{c} \frac{I\left(C^s_{m*} = C^s_m \cap m+1 \neq M\right) + I\left(C^s_{m*} = C^s_{m+1} \cap m \neq 1\right)}{K-2} \\ + \frac{I\left(C^s_{m*} = C^s_m \cap m+1 = M\right) + I\left(C^s_{m*} = C^s_{m+1} \cap m = 1\right)}{K-1} \end{array} \right),$$

where

$$\Psi_{m^*}=\left(\beta_{C^s_{m^*}}+\frac{\kappa_{C^s_{m^*}}\mu^2_{0C^s_{m^*}}+\sum_{i=L_0+\ldots+L_{m-1}+1}^{L_1+\ldots+L_{m^*}}D_i^2-\frac{\left(\kappa_{C^s_{m^*}}\mu_{0C^s_{m^*}}+\sum_{i=L_0+\ldots+L_{m-1}+1}^{L_1+\ldots+L_{m^*}}D_i\right)^2}{\kappa_{C^s_{m^*}}+L_{m^*}}}{2}\right)^{\alpha_{C^s_{m^*}}+\frac{L_{m^*}}{2}}$$

$$\Psi_{m}=\left(\beta_{C^s_{m}}+\frac{\kappa_{C^s_{m}}\mu^2_{0C^s_{m}}+\sum_{i=L_0+\ldots+L_{m-1}+1}^{L_1+\ldots+L_{m}}D_i^2-\frac{\left(\kappa_{C^s_{m}}\mu_{0C^s_{m}}+\sum_{i=L_0+\ldots+L_{m-1}+1}^{L_1+\ldots+L_{m}}D_i\right)^2}{\kappa_{C^s_{m}}+L_{m}}}{2}\right)^{\alpha_{C^s_{m}}+\frac{L_{m}}{2}}$$

$$\Psi_{m+1}=\left(\beta_{C^s_{m+1}}+\frac{\kappa_{C^s_{m+1}}\mu^2_{0C^s_{m+1}}+\sum_{i=L_0+\ldots+L_{m}+1}^{L_1+\ldots+L_{m+1}}D_i^2-\frac{\left(\kappa_{C^s_{m+1}}\mu_{0C^s_{m+1}}+\sum_{i=L_0+\ldots+L_{m}+1}^{L_1+\ldots+L_{m+1}}D_i\right)^2}{\kappa_{C^s_{m+1}}+L_{m+1}}}{2}\right)^{\alpha_{C^s_{m+1}}+\frac{L_{m+1}}{2}}$$

$$A_S = \frac{p\left(\underset{\sim}{\mathbf{B}}_{(After)}, \underset{\sim}{\mathbf{C}}_{\mathbf{1}}, \ldots, \underset{\sim}{\mathbf{C}}_{\mathbf{m-1}}, \underset{\sim}{\mathbf{C}}_{\mathbf{m*}}, \underset{\sim}{\mathbf{C}}_{\mathbf{m**}}, \underset{\sim}{\mathbf{C}}_{\mathbf{m+1}}, \ldots, \underset{\sim}{\mathbf{C}}_{\mathbf{M}} \middle| \underset{\sim}{\mathbf{D}}\right)}{p\left(\underset{\sim}{\mathbf{B}}_{(Before)}, \underset{\sim}{\mathbf{C}}_{\mathbf{1}}, \ldots, \underset{\sim}{\mathbf{C}}_{\mathbf{m-1}}, \underset{\sim}{\mathbf{C}}_{\mathbf{m}}, \underset{\sim}{\mathbf{C}}_{\mathbf{m+1}}, \ldots, \underset{\sim}{\mathbf{C}}_{\mathbf{M}} \middle| \underset{\sim}{\mathbf{D}}\right)}$$

$$\times \frac{q\left(\underset{\sim}{\mathbf{B}}_{(Before)}, \underset{\sim}{\mathbf{C}}_{\mathbf{1}}, \ldots, \underset{\sim}{\mathbf{C}}_{\mathbf{M}} \middle| \underset{\sim}{\mathbf{B}}_{(After)}, \underset{\sim}{\mathbf{C}}_{\mathbf{1}}, \ldots, \underset{\sim}{\mathbf{C}}_{\mathbf{m-1}}, \underset{\sim}{\mathbf{C}}_{\mathbf{m*}}, \underset{\sim}{\mathbf{C}}_{\mathbf{m**}}, \underset{\sim}{\mathbf{C}}_{\mathbf{m+1}}, \ldots, \underset{\sim}{\mathbf{C}}_{\mathbf{M}}\right) \times p\left(\underset{\sim}{\mathbf{u}}\right)}{q\left(\underset{\sim}{\mathbf{B}}_{(After)}, \underset{\sim}{\mathbf{C}}_{\mathbf{1}}, \ldots, \underset{\sim}{\mathbf{C}}_{\mathbf{m-1}}, \underset{\sim}{\mathbf{C}}_{\mathbf{m*}}, \underset{\sim}{\mathbf{C}}_{\mathbf{m**}}, \underset{\sim}{\mathbf{C}}_{\mathbf{m+1}}, \ldots, \underset{\sim}{\mathbf{C}}_{\mathbf{M}} \middle| \underset{\sim}{\mathbf{B}}_{(Before)}, \underset{\sim}{\mathbf{C}}_{\mathbf{1}}, \ldots, \underset{\sim}{\mathbf{C}}_{\mathbf{M}}\right)}$$

$$\times \left| \frac{\partial\left(\underset{\sim}{\mathbf{B}}_{(After)}, \underset{\sim}{\mathbf{C}}_{\mathbf{1}}, \ldots, \underset{\sim}{\mathbf{C}}_{\mathbf{m-1}}, \underset{\sim}{\mathbf{C}}_{\mathbf{m*}}, \underset{\sim}{\mathbf{C}}_{\mathbf{m**}}, \underset{\sim}{\mathbf{C}}_{\mathbf{m+1}}, \ldots, \underset{\sim}{\mathbf{C}}_{\mathbf{M}}\right)}{\partial\left(\underset{\sim}{\mathbf{B}}_{(Before)}, \underset{\sim}{\mathbf{C}}_{\mathbf{1}}, \ldots, \underset{\sim}{\mathbf{C}}_{\mathbf{M}}, \underset{\sim}{\mathbf{u}}\right)} \right|$$

$$= \frac{\left\{ \frac{\sqrt{\kappa_{C^s_{m*}}}}{\sqrt{\kappa_{C^s_{m*}} + L_{m*}}} \frac{\Gamma\left(\alpha_{C^s_{m*}} + \frac{L_{m*}}{2}\right)}{\Gamma\left(\alpha_{C^s_{m*}}\right)} \frac{\beta_{C^s_{m*}}^{\alpha_{C^s_{m*}}}}{\Psi_{m*}} \times \frac{\sqrt{\kappa_{C^s_{m**}}}}{\sqrt{\kappa_{C^s_{m**}} + L_{m**}}} \frac{\Gamma\left(\alpha_{C^s_{m**}} + \frac{L_{m**}}{2}\right)}{\Gamma\left(\alpha_{C^s_{m**}}\right)} \frac{\beta_{C^s_{m**}}^{\alpha_{C^s_{m**}}}}{\Psi_{m**}} \right\}}{\frac{\sqrt{\kappa_{C^s_{m}}}}{\sqrt{\kappa_{C^s_{m}} + L_{m}}} \frac{\Gamma\left(\alpha_{C^s_{m}} + \frac{L_{m}}{2}\right)}{\Gamma\left(\alpha_{C^s_{m}}\right)} \frac{\beta_{C^s_{m}}^{\alpha_{C^s_{m}}}}{\Psi_{m}}}$$

$$\times \frac{\left\{ \prod_{k=1}^{K} \frac{\Gamma\left(C_{1k(After)} + w_{0k}\right)}{\Gamma\left(w_{0k}\right)} \right\} \times \prod_{k'=1}^{K} \left\{ \frac{1}{\Gamma\left(1 + n_{k'(After)}\right)} \prod_{\substack{k=1 \\ k \neq k'}}^{K} \frac{\Gamma\left(n_{k'k(After)} + \frac{w_{0k}}{1 - w_{0k'}}\right)}{\Gamma\left(\frac{w_{0k}}{1 - w_{0k'}}\right)} \right\}}{\left\{ \prod_{k=1}^{K} \frac{\Gamma\left(C_{1k(Before)} + w_{0k}\right)}{\Gamma\left(w_{0k}\right)} \right\} \times \prod_{k'=1}^{K} \left\{ \frac{1}{\Gamma\left(1 + n_{k'(Before)}\right)} \prod_{\substack{k=1 \\ k \neq k'}}^{K} \frac{\Gamma\left(n_{k'k(Before)} + \frac{w_{0k}}{1 - w_{0k'}}\right)}{\Gamma\left(\frac{w_{0k}}{1 - w_{0k'}}\right)} \right\}} \times \frac{\lambda}{1 - \lambda}$$

$$\times \left(\begin{array}{l} (K-2) \times \left(I\left(C^s_{m*} = C^s_m \cap m+1 \neq M\right) + I\left(C^s_{m**} = C^s_m \cap m \neq 1\right) \right) \\ + (K-1) \times \left(I\left(C^s_{m*} = C^s_m \cap m+1 = M\right) + I\left(C^s_{m**} = C^s_m \cap m = 1\right) \right) \end{array} \right),$$

where

$$\Psi_{m*} = \left(\beta_{C^s_{m*}} + \frac{\kappa_{C^s_{m*}} \mu^2_{0C^s_{m*}} + \sum_{i=L_0+\ldots+L_{m-1}+1}^{L_1+\ldots+L_{m*}} D_i^2 - \frac{\left(\kappa_{C^s_{m*}} \mu_{0C^s_{m*}} + \sum_{i=L_0+\ldots+L_{m-1}+1}^{L_1+\ldots+L_{m*}} D_i \right)^2}{\kappa_{C^s_{m*}} + L_{m*}}}{2} \right)^{\alpha_{C^s_{m*}} + \frac{L_{m*}}{2}}$$

$$\Psi_{m^{**}}=\left(\beta_{C^s_{m^{**}}}+\frac{\kappa_{C^s_{m^{**}}}\mu^2_{0C^s_{m^{**}}}+\sum_{i=L_0+\ldots+L_{m-1}+L_{m^*}+1}^{L_1+\ldots+L_{m^*}+L_{m^{**}}}D_i^2-\frac{\left(\kappa_{C^s_{m^{**}}}\mu_{0C^s_{m^{**}}}+\sum_{i=L_0+\ldots+L_{m-1}+L_{m^*}+1}^{L_1+\ldots+L_{m^*}+L_{m^{**}}}D_i\right)^2}{\kappa_{C^s_{m^{**}}}+L_{m^{**}}}}{2}\right)^{\alpha_{C^s_{m^{**}}}+\frac{L_{m^{**}}}{2}}$$

$$\Psi_m=\left(\beta_{C^s_m}+\frac{\kappa_{C^s_m}\mu^2_{0C^s_m}+\sum_{i=L_0+\ldots+L_{m-1}+1}^{L_1+\ldots+L_m}D_i^2-\frac{\left(\kappa_{C^s_m}\mu_{0C^s_m}+\sum_{i=L_0+\ldots+L_{m-1}+1}^{L_1+\ldots+L_m}D_i\right)^2}{\kappa_{C^s_m}+L_m}}{2}\right)^{\alpha_{C^s_m}+\frac{L_m}{2}}$$

Second, double merge and trifid are opposite strategies with accepted probabilities $\min\{1,A_{M2.1}\}/\min\{1,A_{M2.2}\}$ and $\min\{1,A_T\}$, respectively. In the double merge strategy, we merge two neighboring regions in the first step. If the proposed state of the new region is equal to that of the adjacent region, then we merge these regions again. Based on the procedure introduced in the above subsection, the transition density function is calculated. Moreover, the variables $\underset{\sim}{\mathbf{u}}_1$ and $\underset{\sim}{\mathbf{u}}_2$ should be fixed, and their joint probability is set to 1. The Jacobian of the transformation is equal to 1.

For the reverse move trifid, we randomly select two window boundaries that belong to region *m* to change the value. Then, the region is split into *m**, *m***, and *m****. The states of the three updated regions were previously illustrated, and the transition density function could be derived. The variables $\underset{\sim}{\mathbf{u}}_1$ and $\underset{\sim}{\mathbf{u}}_2$ should also be fixed with joint probability 1. The Jacobian of the transformation is equal to 1.

$A_{M2.1}$, $A_{M2.2}$ and A_T are calculated as follows:

$$A_{M2.1} = \frac{p\left(\underset{\sim}{\mathbf{B}}_{(After)}, \underset{\sim}{\mathbf{C}}_{\mathbf{1}}, \ldots, \underset{\sim}{\mathbf{C}}_{\mathbf{m-1}}, \underset{\sim}{\mathbf{C}}_{\mathbf{m**}}, \underset{\sim}{\mathbf{C}}_{\mathbf{m+3}}, \ldots, \underset{\sim}{\mathbf{C}}_{\mathbf{M}} \middle| \underset{\sim}{\mathbf{D}}\right)}{p\left(\underset{\sim}{\mathbf{B}}_{(Before)}, \underset{\sim}{\mathbf{C}}_{\mathbf{1}}, \ldots, \underset{\sim}{\mathbf{C}}_{\mathbf{m-1}}, \underset{\sim}{\mathbf{C}}_{\mathbf{m}}, \underset{\sim}{\mathbf{C}}_{\mathbf{m+1}}, \ldots, \underset{\sim}{\mathbf{C}}_{\mathbf{M}} \middle| \underset{\sim}{\mathbf{D}}\right)}$$

$$\times \frac{q\left(\underset{\sim}{\mathbf{B}}_{(Before)}, \underset{\sim}{\mathbf{C}}_{\mathbf{1}}, \ldots, \underset{\sim}{\mathbf{C}}_{\mathbf{M}} \middle| \underset{\sim}{\mathbf{B}}_{(After)}, \underset{\sim}{\mathbf{C}}_{\mathbf{1}}, \ldots, \underset{\sim}{\mathbf{C}}_{\mathbf{m-1}}, \underset{\sim}{\mathbf{C}}_{\mathbf{m**}}, \underset{\sim}{\mathbf{C}}_{\mathbf{m+3}}, \ldots, \underset{\sim}{\mathbf{C}}_{\mathbf{M}}\right) \times p\left(\underset{\sim}{\mathbf{u}}_1, \underset{\sim}{\mathbf{u}}_2\right)}{q\left(\underset{\sim}{\mathbf{B}}_{(After)}, \underset{\sim}{\mathbf{C}}_{\mathbf{1}}, \ldots, \underset{\sim}{\mathbf{C}}_{\mathbf{m-1}}, \underset{\sim}{\mathbf{C}}_{\mathbf{m**}}, \underset{\sim}{\mathbf{C}}_{\mathbf{m+3}}, \ldots, \underset{\sim}{\mathbf{C}}_{\mathbf{M}} \middle| \underset{\sim}{\mathbf{B}}_{(Before)}, \underset{\sim}{\mathbf{C}}_{\mathbf{1}}, \ldots, \underset{\sim}{\mathbf{C}}_{\mathbf{M}}\right)}$$

$$\times \left| \frac{\partial\left(\underset{\sim}{\mathbf{B}}_{(After)}, \underset{\sim}{\mathbf{C}}_{\mathbf{1}}, \ldots, \underset{\sim}{\mathbf{C}}_{\mathbf{m-1}}, \underset{\sim}{\mathbf{C}}_{\mathbf{m**}}, \underset{\sim}{\mathbf{C}}_{\mathbf{m+3}}, \ldots, \underset{\sim}{\mathbf{C}}_{\mathbf{M}}, \underset{\sim}{\mathbf{u}}_1, \underset{\sim}{\mathbf{u}}_2\right)}{\partial\left(\underset{\sim}{\mathbf{B}}_{(Before)}, \underset{\sim}{\mathbf{C}}_{\mathbf{1}}, \ldots, \underset{\sim}{\mathbf{C}}_{\mathbf{M}}\right)} \right|$$

$$= \frac{\dfrac{\sqrt{\kappa_{C^s_{m**}}}}{\sqrt{\kappa_{C^s_{m**}} + L_{m**}}} \dfrac{\Gamma\left(\alpha_{C^s_{m**}} + \frac{L_{m**}}{2}\right)}{\Gamma\left(\alpha_{C^s_{m**}}\right)} \dfrac{\beta_{C^s_{m**}}^{\alpha_{C^s_{m**}}}}{\Psi_{m**}}}{\left\{ \begin{array}{l} \dfrac{\sqrt{\kappa_{C^s_{m}}}}{\sqrt{\kappa_{C^s_{m}} + L_{m}}} \dfrac{\Gamma\left(\alpha_{C^s_{m}} + \frac{L_{m}}{2}\right)}{\Gamma\left(\alpha_{C^s_{m}}\right)} \dfrac{\beta_{C^s_{m}}^{\alpha_{C^s_{m}}}}{\Psi_{m}} \\ \times \dfrac{\sqrt{\kappa_{C^s_{m+1}}}}{\sqrt{\kappa_{C^s_{m+1}} + L_{m+1}}} \dfrac{\Gamma\left(\alpha_{C^s_{m+1}} + \frac{L_{m+1}}{2}\right)}{\Gamma\left(\alpha_{C^s_{m+1}}\right)} \dfrac{\beta_{C^s_{m+1}}^{\alpha_{C^s_{m+1}}}}{\Psi_{m+1}} \\ \times \dfrac{\sqrt{\kappa_{C^s_{m+2}}}}{\sqrt{\kappa_{C^s_{m+2}} + L_{m+2}}} \dfrac{\Gamma\left(\alpha_{C^s_{m+2}} + \frac{L_{m+2}}{2}\right)}{\Gamma\left(\alpha_{C^s_{m+2}}\right)} \dfrac{\beta_{C^s_{m+2}}^{\alpha_{C^s_{m+2}}}}{\Psi_{m+2}} \end{array} \right\}} \times \frac{(1-\lambda)^2}{\lambda^2} \times \frac{4}{(I-2)\times(K-1)}$$

$$\times \frac{\left\{\prod_{k=1}^{K} \dfrac{\Gamma\left(C_{1k(After)} + w_{0k}\right)}{\Gamma\left(w_{0k}\right)}\right\} \times \prod_{k'=1}^{K} \left\{ \dfrac{1}{\Gamma\left(1 + n_{k'(After)}\right)} \prod_{\substack{k=1 \\ k \neq k'}}^{K} \dfrac{\Gamma\left(n_{k'k(After)} + \frac{w_{0k}}{1-w_{0k'}}\right)}{\Gamma\left(\frac{w_{0k}}{1-w_{0k'}}\right)} \right\}}{\left\{\prod_{k=1}^{K} \dfrac{\Gamma\left(C_{1k(Before)} + w_{0k}\right)}{\Gamma\left(w_{0k}\right)}\right\} \times \prod_{k'=1}^{K} \left\{ \dfrac{1}{\Gamma\left(1 + n_{k'(Before)}\right)} \prod_{\substack{k=1 \\ k \neq k'}}^{K} \dfrac{\Gamma\left(n_{k'k(Before)} + \frac{w_{0k}}{1-w_{0k'}}\right)}{\Gamma\left(\frac{w_{0k}}{1-w_{0k'}}\right)} \right\}}$$

where

$$\Psi_{m^{**}} = \left(\beta_{C^s_{m^{**}}} + \frac{\kappa_{C^s_{m^{**}}} \mu^2_{0C^s_{m^{**}}} + \sum_{i=L_0+\ldots+L_{m-1}+1}^{L_1+\ldots+L_{m^{**}}} D_i^2 - \frac{\left(\kappa_{C^s_{m^{**}}} \mu_{0C^s_{m^{**}}} + \sum_{i=L_0+\ldots+L_{m-1}+1}^{L_1+\ldots+L_{m^{**}}} D_i \right)^2}{\kappa_{C^s_{m^{**}}} + L_{m^{**}}}}{2} \right)^{\alpha_{C^s_{m^{**}}} + \frac{L_{m^{**}}}{2}}$$

$$\Psi_{m} = \left(\beta_{C^s_{m}} + \frac{\kappa_{C^s_{m}} \mu^2_{0C^s_{m}} + \sum_{i=L_0+\ldots+L_{m-1}+1}^{L_1+\ldots+L_{m}} D_i^2 - \frac{\left(\kappa_{C^s_{m}} \mu_{0C^s_{m}} + \sum_{i=L_0+\ldots+L_{m-1}+1}^{L_1+\ldots+L_{m}} D_i \right)^2}{\kappa_{C^s_{m}} + L_{m}}}{2} \right)^{\alpha_{C^s_{m}} + \frac{L_{m}}{2}}$$

$$\Psi_{m+1} = \left(\beta_{C^s_{m+1}} + \frac{\kappa_{C^s_{m+1}} \mu^2_{0C^s_{m+1}} + \sum_{i=L_0+\ldots+L_{m}+1}^{L_1+\ldots+L_{m+1}} D_i^2 - \frac{\left(\kappa_{C^s_{m+1}} \mu_{0C^s_{m+1}} + \sum_{i=L_0+\ldots+L_{m}+1}^{L_1+\ldots+L_{m+1}} D_i \right)^2}{\kappa_{C^s_{m+1}} + L_{m+1}}}{2} \right)^{\alpha_{C^s_{m+1}} + \frac{L_{m+1}}{2}}$$

$$\Psi_{m+2} = \left(\beta_{C^s_{m+2}} + \frac{\kappa_{C^s_{m+2}} \mu^2_{0C^s_{m+2}} + \sum_{i=L_0+\ldots+L_{m+1}+1}^{L_1+\ldots+L_{m+2}} D_i^2 - \frac{\left(\kappa_{C^s_{m+2}} \mu_{0C^s_{m+2}} + \sum_{i=L_0+\ldots+L_{m+1}+1}^{L_1+\ldots+L_{m+2}} D_i \right)^2}{\kappa_{C^s_{m+2}} + L_{m+2}}}{2} \right)^{\alpha_{C^s_{m+2}} + \frac{L_{m+2}}{2}}$$

$$A_{M\,2.2}=\frac{p\left(\underset{\sim}{\mathbf{B}}_{(After)},\underset{\sim}{\mathbf{C}}_{\mathbf{1}},\ldots,\underset{\sim}{\mathbf{C}}_{\mathbf{m\text{-}2}},\underset{\sim}{\mathbf{C}}_{\mathbf{m\text{-}1^{**}}},\underset{\sim}{\mathbf{C}}_{\mathbf{m+2}},\ldots,\underset{\sim}{\mathbf{C}}_{\mathbf{M}}\,\middle|\,\underset{\sim}{\mathbf{D}}\right)}{p\left(\underset{\sim}{\mathbf{B}}_{(Before)},\underset{\sim}{\mathbf{C}}_{\mathbf{1}},\ldots,\underset{\sim}{\mathbf{C}}_{\mathbf{m\text{-}1}},\underset{\sim}{\mathbf{C}}_{\mathbf{m}},\underset{\sim}{\mathbf{C}}_{\mathbf{m+1}},\ldots,\underset{\sim}{\mathbf{C}}_{\mathbf{M}}\,\middle|\,\underset{\sim}{\mathbf{D}}\right)}$$

$$\times\frac{q\left(\underset{\sim}{\mathbf{B}}_{(Before)},\underset{\sim}{\mathbf{C}}_{\mathbf{1}},\ldots,\underset{\sim}{\mathbf{C}}_{\mathbf{M}}\,\middle|\,\underset{\sim}{\mathbf{B}}_{(After)},\underset{\sim}{\mathbf{C}}_{\mathbf{1}},\ldots,\underset{\sim}{\mathbf{C}}_{\mathbf{m\text{-}2}},\underset{\sim}{\mathbf{C}}_{\mathbf{m\text{-}1^{**}}},\underset{\sim}{\mathbf{C}}_{\mathbf{m+2}},\ldots,\underset{\sim}{\mathbf{C}}_{\mathbf{M}}\right)\times p\left(\underset{\sim}{\mathbf{u}}_1,\underset{\sim}{\mathbf{u}}_2\right)}{q\left(\underset{\sim}{\mathbf{B}}_{(After)},\underset{\sim}{\mathbf{C}}_{\mathbf{1}},\ldots,\underset{\sim}{\mathbf{C}}_{\mathbf{m\text{-}2}},\underset{\sim}{\mathbf{C}}_{\mathbf{m\text{-}1^{**}}},\underset{\sim}{\mathbf{C}}_{\mathbf{m+2}},\ldots,\underset{\sim}{\mathbf{C}}_{\mathbf{M}}\,\middle|\,\underset{\sim}{\mathbf{B}}_{(Before)},\underset{\sim}{\mathbf{C}}_{\mathbf{1}},\ldots,\underset{\sim}{\mathbf{C}}_{\mathbf{M}}\right)}$$

$$\times\left|\frac{\partial\left(\underset{\sim}{\mathbf{B}}_{(After)},\underset{\sim}{\mathbf{C}}_{\mathbf{1}},\ldots,\underset{\sim}{\mathbf{C}}_{\mathbf{m\text{-}2}},\underset{\sim}{\mathbf{C}}_{\mathbf{m\text{-}1^{**}}},\underset{\sim}{\mathbf{C}}_{\mathbf{m+2}},\ldots,\underset{\sim}{\mathbf{C}}_{\mathbf{M}},\underset{\sim}{\mathbf{u}}_1,\underset{\sim}{\mathbf{u}}_2\right)}{\partial\left(\underset{\sim}{\mathbf{B}}_{(Before)},\underset{\sim}{\mathbf{C}}_{\mathbf{1}},\ldots,\underset{\sim}{\mathbf{C}}_{\mathbf{M}}\right)}\right|$$

$$=\frac{\dfrac{\sqrt{\kappa_{C^s_{m-1^{**}}}}}{\sqrt{\kappa_{C^s_{m-1^{**}}}+L_{m-1^{**}}}}\dfrac{\Gamma\left(\alpha_{C^s_{m-1^{**}}}+\dfrac{L_{m-1^{**}}}{2}\right)}{\Gamma\left(\alpha_{C^s_{m-1^{**}}}\right)}\dfrac{\beta_{C^s_{m-1^{**}}}^{\alpha_{C^s_{m\text{-}1^{**}}}}}{\Psi_{m-1^{**}}}}{\left\{\begin{array}{l}\dfrac{\sqrt{\kappa_{C^s_{m\text{-}1}}}}{\sqrt{\kappa_{C^s_{m\text{-}1}}+L_{m\text{-}1}}}\dfrac{\Gamma\left(\alpha_{C^s_{m\text{-}1}}+\dfrac{L_{m\text{-}1}}{2}\right)}{\Gamma\left(\alpha_{C^s_{m\text{-}1}}\right)}\dfrac{\beta_{C^s_{m\text{-}1}}^{\alpha_{C^s_{m\text{-}1}}}}{\Psi_{m-1}}\\ \times\dfrac{\sqrt{\kappa_{C^s_m}}}{\sqrt{\kappa_{C^s_m}+L_m}}\dfrac{\Gamma\left(\alpha_{C^s_m}+\dfrac{L_m}{2}\right)}{\Gamma\left(\alpha_{C^s_m}\right)}\dfrac{\beta_{C^s_m}^{\alpha_{C^s_m}}}{\Psi_m}\\ \times\dfrac{\sqrt{\kappa_{C^s_{m+1}}}}{\sqrt{\kappa_{C^s_{m+1}}+L_{m+1}}}\dfrac{\Gamma\left(\alpha_{C^s_{m+1}}+\dfrac{L_{m+1}}{2}\right)}{\Gamma\left(\alpha_{C^s_{m+1}}\right)}\dfrac{\beta_{C^s_{m+1}}^{\alpha_{C^s_{m+1}}}}{\Psi_{m+1}}\end{array}\right\}}\times\frac{(1-\lambda)^2}{\lambda^2}\times\frac{4}{(I-2)\times(K-1)}$$

$$\times\frac{\left\{\prod_{k=1}^{K}\dfrac{\Gamma\left(C_{1k(After)}+w_{0k}\right)}{\Gamma\left(w_{0k}\right)}\right\}\times\prod_{k'=1}^{K}\left\{\dfrac{1}{\Gamma\left(1+n_{k'(After)}\right)}\prod_{\substack{k=1\\k\neq k'}}^{K}\dfrac{\Gamma\left(n_{k'k(After)}+\dfrac{w_{0k}}{1-w_{0k'}}\right)}{\Gamma\left(\dfrac{w_{0k}}{1-w_{0k'}}\right)}\right\}}{\times\left\{\prod_{k=1}^{K}\dfrac{\Gamma\left(C_{1k(Before)}+W_{0k}\right)}{\Gamma\left(W_{0k}\right)}\right\}\times\prod_{k'=1}^{K}\left\{\dfrac{1}{\Gamma\left(1+n_{k'(Before)}\right)}\prod_{\substack{k=1\\k\neq k'}}^{K}\dfrac{\Gamma\left(n_{k'k(Before)}+\dfrac{w_{0k}}{1-w_{0k'}}\right)}{\Gamma\left(\dfrac{w_{0k}}{1-w_{0k'}}\right)}\right\}},$$

where $$\Psi_{m-1^{**}}=\left(\beta_{C^s_{m-1^{**}}}+\frac{\kappa_{C^s_{m-1^{**}}}\mu^2_{0C^s_{m-1^{**}}}+\sum_{i=L_0+\ldots+L_{m-2}+1}^{L_1+\ldots+L_{m-1^{**}}}D_i^2-\frac{\left(\kappa_{C^s_{m-1^{**}}}\mu_{0C^s_{m-1^{**}}}+\sum_{i=L_0+\ldots+L_{m-2}+1}^{L_1+\ldots+L_{m-1^{**}}}D_i\right)^2}{\kappa_{C^s_{m-1^{**}}}+L_{m-1^{**}}}}{2}\right)^{\alpha_{C^s_{m-1^{**}}}+\frac{L_{m-1^{**}}}{2}}$$

$$\Psi_{m-1}=\left(\beta_{C^s_{m\text{-}1}}+\frac{\kappa_{C^s_{m\text{-}1}}\mu^2_{0C^s_{m\text{-}1}}+\sum_{i=L_0+\ldots+L_{m\text{-}2}+1}^{L_1+\ldots+L_{m\text{-}1}}D_i^2-\frac{\left(\kappa_{C^s_{m\text{-}1}}\mu_{0C^s_{m\text{-}1}}+\sum_{i=L_0+\ldots+L_{m\text{-}2}+1}^{L_1+\ldots+L_{m\text{-}1}}D_i\right)^2}{\kappa_{C^s_{m\text{-}1}}+L_{m\text{-}1}}}{2}\right)^{\alpha_{C^s_{m\text{-}1}}+\frac{L_{m\text{-}1}}{2}}$$

$$\Psi_{m}=\left(\beta_{C^s_{m}}+\frac{\kappa_{C^s_{m}}\mu^2_{0C^s_{m}}+\sum_{i=L_0+\ldots+L_{m-1}+1}^{L_1+\ldots+L_{m}}D_i^2-\frac{\left(\kappa_{C^s_{m}}\mu_{0C^s_{m}}+\sum_{i=L_0+\ldots+L_{m-1}+1}^{L_1+\ldots+L_{m}}D_i\right)^2}{\kappa_{C^s_{m}}+L_{m}}}{2}\right)^{\alpha_{C^s_{m}}+\frac{L_{m}}{2}}$$

$$\Psi_{m+1}=\left(\beta_{C^s_{m+1}}+\frac{\kappa_{C^s_{m+1}}\mu^2_{0C^s_{m+1}}+\sum_{i=L_0+\ldots+L_{m}+1}^{L_1+\ldots+L_{m+1}}D_i^2-\frac{\left(\kappa_{C^s_{m+1}}\mu_{0C^s_{m+1}}+\sum_{i=L_0+\ldots+L_{m}+1}^{L_1+\ldots+L_{m+1}}D_i\right)^2}{\kappa_{C^s_{m+1}}+L_{m+1}}}{2}\right)^{\alpha_{C^s_{m+1}}+\frac{L_{m+1}}{2}}$$

$$A_T = \frac{p\left(\underset{\sim}{\mathbf{B}}_{(After)}, \underset{\sim}{\mathbf{C}}_{\mathbf{1}}, \ldots, \underset{\sim}{\mathbf{C}}_{\mathbf{m-1}}, \underset{\sim}{\mathbf{C}}_{\mathbf{m*}}, \underset{\sim}{\mathbf{C}}_{\mathbf{m**}}, \underset{\sim}{\mathbf{C}}_{\mathbf{m***}}, \underset{\sim}{\mathbf{C}}_{\mathbf{m+1}}, \ldots, \underset{\sim}{\mathbf{C}}_{\mathbf{M}} \middle| \underset{\sim}{\mathbf{D}}\right)}{p\left(\underset{\sim}{\mathbf{B}}_{(Before)}, \underset{\sim}{\mathbf{C}}_{\mathbf{1}}, \ldots, \underset{\sim}{\mathbf{C}}_{\mathbf{m-1}}, \underset{\sim}{\mathbf{C}}_{\mathbf{m}}, \underset{\sim}{\mathbf{C}}_{\mathbf{m+1}}, \ldots, \underset{\sim}{\mathbf{C}}_{\mathbf{M}} \middle| \underset{\sim}{\mathbf{D}}\right)}$$

$$\times \frac{q\left(\underset{\sim}{\mathbf{B}}_{(Before)}, \underset{\sim}{\mathbf{C}}_{\mathbf{1}}, \ldots, \underset{\sim}{\mathbf{C}}_{\mathbf{M}} \middle| \underset{\sim}{\mathbf{B}}_{(After)}, \underset{\sim}{\mathbf{C}}_{\mathbf{1}}, \ldots, \underset{\sim}{\mathbf{C}}_{\mathbf{m-1}}, \underset{\sim}{\mathbf{C}}_{\mathbf{m*}}, \underset{\sim}{\mathbf{C}}_{\mathbf{m**}}, \underset{\sim}{\mathbf{C}}_{\mathbf{m***}}, \underset{\sim}{\mathbf{C}}_{\mathbf{m+1}}, \ldots, \underset{\sim}{\mathbf{C}}_{\mathbf{M}}\right) \times p\left(\underset{\sim}{\mathbf{u}}_{\mathbf{1}}, \underset{\sim}{\mathbf{u}}_{\mathbf{2}}\right)}{q\left(\underset{\sim}{\mathbf{B}}_{(After)}, \underset{\sim}{\mathbf{C}}_{\mathbf{1}}, \ldots, \underset{\sim}{\mathbf{C}}_{\mathbf{m-1}}, \underset{\sim}{\mathbf{C}}_{\mathbf{m*}}, \underset{\sim}{\mathbf{C}}_{\mathbf{m**}}, \underset{\sim}{\mathbf{C}}_{\mathbf{m***}}, \underset{\sim}{\mathbf{C}}_{\mathbf{m+1}}, \ldots, \underset{\sim}{\mathbf{C}}_{\mathbf{M}} \middle| \underset{\sim}{\mathbf{B}}_{(Before)}, \underset{\sim}{\mathbf{C}}_{\mathbf{1}}, \ldots, \underset{\sim}{\mathbf{C}}_{\mathbf{M}}\right)}$$

$$\times \left| \frac{\partial\left(\underset{\sim}{\mathbf{B}}_{(After)}, \underset{\sim}{\mathbf{C}}_{\mathbf{1}}, \ldots, \underset{\sim}{\mathbf{C}}_{\mathbf{m-1}}, \underset{\sim}{\mathbf{C}}_{\mathbf{m*}}, \underset{\sim}{\mathbf{C}}_{\mathbf{m**}}, \underset{\sim}{\mathbf{C}}_{\mathbf{m***}}, \underset{\sim}{\mathbf{C}}_{\mathbf{m+1}}, \ldots, \underset{\sim}{\mathbf{C}}_{\mathbf{M}}\right)}{\partial\left(\underset{\sim}{\mathbf{B}}_{(Before)}, \underset{\sim}{\mathbf{C}}_{\mathbf{1}}, \ldots, \underset{\sim}{\mathbf{C}}_{\mathbf{M}}, \underset{\sim}{\mathbf{u}}_{\mathbf{1}}, \underset{\sim}{\mathbf{u}}_{\mathbf{2}}\right)} \right|$$

$$= \frac{\left\{ \begin{array}{l} \frac{\sqrt{\kappa_{C^s_{m*}}}}{\sqrt{\kappa_{C^s_{m*}} + L_{m*}}} \frac{\Gamma\left(\alpha_{C^s_{m*}} + \frac{L_{m*}}{2}\right)}{\Gamma\left(\alpha_{C^s_{m*}}\right)} \frac{\beta_{C^s_{m*}}^{\alpha_{C^s_{m*}}}}{\Psi_{m*}} \\ \frac{\sqrt{\kappa_{C^s_{m**}}}}{\sqrt{\kappa_{C^s_{m**}} + L_{m**}}} \frac{\Gamma\left(\alpha_{C^s_{m**}} + \frac{L_{m**}}{2}\right)}{\Gamma\left(\alpha_{C^s_{m**}}\right)} \frac{\beta_{C^s_{m**}}^{\alpha_{C^s_{m**}}}}{\Psi_{m**}} \\ \times \frac{\sqrt{\kappa_{C^s_{m***}}}}{\sqrt{\kappa_{C^s_{m***}} + L_{m***}}} \frac{\Gamma\left(\alpha_{C^s_{m***}} + \frac{L_{m***}}{2}\right)}{\Gamma\left(\alpha_{C^s_{m***}}\right)} \frac{\beta_{C^s_{m***}}^{\alpha_{C^s_{m***}}}}{\Psi_{m***}} \end{array} \right\}}{\frac{\sqrt{\kappa_{C^s_{m}}}}{\sqrt{\kappa_{C^s_{m}} + L_{m}}} \frac{\Gamma\left(\alpha_{C^s_{m}} + \frac{L_{m}}{2}\right)}{\Gamma\left(\alpha_{C^s_{m}}\right)} \frac{\beta_{C^s_{m}}^{\alpha_{C^s_{m}}}}{\Psi_{m}}}$$

$$\times \frac{\left\{\prod_{k=1}^{K} \frac{\Gamma\left(C_{1k(After)} + w_{0k}\right)}{\Gamma\left(w_{0k}\right)}\right\} \times \prod_{k'=1}^{K} \left\{ \frac{1}{\Gamma\left(1 + n_{k'(After)}\right)} \prod_{\substack{k=1 \\ k \neq k'}}^{K} \frac{\Gamma\left(n_{k'k(After)} + \frac{w_{0k}}{1 - w_{0k'}}\right)}{\Gamma\left(\frac{w_{0k}}{1 - w_{0k'}}\right)} \right\}}{\left\{\prod_{k=1}^{K} \frac{\Gamma\left(C_{1k(Before)} + w_{0k}\right)}{\Gamma\left(w_{0k}\right)}\right\} \times \prod_{k'=1}^{K} \left\{ \frac{1}{\Gamma\left(1 + n_{k'(Before)}\right)} \prod_{\substack{k=1 \\ k \neq k'}}^{K} \frac{\Gamma\left(n_{k'k(Before)} + \frac{w_{0k}}{1 - w_{0k'}}\right)}{\Gamma\left(\frac{w_{0k}}{1 - w_{0k'}}\right)} \right\}}$$

$$\times \frac{\lambda^2}{\left(1-\lambda\right)^2} \times \frac{\left(K-1\right) \times \left(I-2\right)}{4},$$

where

$$\Psi_{m^*}=\left(\beta_{C^s_{m^*}}+\frac{\kappa_{C^s_{m^*}}\mu^2_{0C^s_{m^*}}+\sum_{i=L_0+\ldots+L_{m-1}+1}^{L_1+\ldots+L_{m^*}}D_i^2-\frac{\left(\kappa_{C^s_{m^*}}\mu_{0C^s_{m^*}}+\sum_{i=L_0+\ldots+L_{m-1}+1}^{L_1+\ldots+L_{m^*}}D_i\right)^2}{\kappa_{C^s_{m^*}}+L_{m^*}}}{2}\right)^{\alpha_{C^s_{m^*}}+\frac{L_{m^*}}{2}}$$

$$\Psi_{m^{**}}=\left(\beta_{C^s_{m^{**}}}+\frac{\kappa_{C^s_{m^{**}}}\mu^2_{0C^s_{m^{**}}}+\sum_{i=L_0+\ldots+L_{m-1}+L_{m^*}+1}^{L_1+\ldots+L_{m^*}+L_{m^{**}}}D_i^2-\frac{\left(\kappa_{C^s_{m^{**}}}\mu_{0C^s_{m^{**}}}+\sum_{i=L_0+\ldots+L_{m-1}+L_{m^*}+1}^{L_1+\ldots+L_{m^*}+L_{m^{**}}}D_i\right)^2}{\kappa_{C^s_{m^{**}}}+L_{m^{**}}}}{2}\right)^{\alpha_{C^s_{m^{**}}}+\frac{L_{m^{**}}}{2}}$$

$$\Psi_{m^{***}}=\left(\beta_{C^s_{m^{***}}}+\frac{\kappa_{C^s_{m^{***}}}\mu^2_{0C^s_{m^{***}}}+\sum_{i=L_0+\ldots+L_{m-1}+L_{m^*}+L_{m^{**}}+1}^{L_1+\ldots+L_{m^*}+L_{m^{**}}+L_{m^{**}}}D_i^2-\frac{\left(\kappa_{C^s_{m^{***}}}\mu_{0C^s_{m^{***}}}+\sum_{i=L_0+\ldots+L_{m-1}+L_{m^*}+L_{m^{**}}+1}^{L_1+\ldots+L_{m^*}+L_{m^{**}}+L_{m^{***}}}D_i\right)^2}{\kappa_{C^s_{m^{***}}}+L_{m^{***}}}}{2}\right)^{\alpha_{C^s_{m^{***}}}+\frac{L_{m^{***}}}{2}}$$

$$\Psi_{m}=\left(\beta_{C^s_{m}}+\frac{\kappa_{C^s_{m}}\mu^2_{0C^s_{m}}+\sum_{i=L_0+\ldots+L_{m-1}+1}^{L_1+\ldots+L_{m}}D_i^2-\frac{\left(\kappa_{C^s_{m}}\mu_{0C^s_{m}}+\sum_{i=L_0+\ldots+L_{m-1}+1}^{L_1+\ldots+L_{m}}D_i\right)^2}{\kappa_{C^s_{m}}+L_{m}}}{2}\right)^{\alpha_{C^s_{m}}+\frac{L_{m}}{2}}$$

Third, the boundary changes to the left and right are opposite strategies with accepted probabilities A_{B-1} and A_{B+1}, respectively. Based on the previously illustrated procedure, the transition density functions are built. Because the parameter dimension should not change in this move, the variable $\underset{\sim}{\mathbf{u}}$ is unnecessary. The Jacobian is equal to 1.

$$A_{B-1} = \frac{p\left(\underset{\sim}{\mathbf{B}}_{(After)}, \underset{\sim}{\mathbf{C}}_1, \underset{\sim}{\mathbf{C}}_2, \ldots, \underset{\sim}{\mathbf{C}}_\mathbf{M} \middle| \underset{\sim}{\mathbf{D}}\right)}{p\left(\underset{\sim}{\mathbf{B}}_{(Before)}, \underset{\sim}{\mathbf{C}}_1, \underset{\sim}{\mathbf{C}}_2, \ldots, \underset{\sim}{\mathbf{C}}_\mathbf{M} \middle| \underset{\sim}{\mathbf{D}}\right)} \times \frac{q\left(\underset{\sim}{\mathbf{B}}_{(Before)}, \underset{\sim}{\mathbf{C}}_1, \underset{\sim}{\mathbf{C}}_2, \ldots, \underset{\sim}{\mathbf{C}}_\mathbf{M} \middle| \underset{\sim}{\mathbf{B}}_{(After)}, \underset{\sim}{\mathbf{C}}_1, \underset{\sim}{\mathbf{C}}_2, \ldots, \underset{\sim}{\mathbf{C}}_\mathbf{M}\right)}{q\left(\underset{\sim}{\mathbf{B}}_{(After)}, \underset{\sim}{\mathbf{C}}_1, \underset{\sim}{\mathbf{C}}_2, \ldots, \underset{\sim}{\mathbf{C}}_\mathbf{M} \middle| \underset{\sim}{\mathbf{B}}_{(Before)}, \underset{\sim}{\mathbf{C}}_1, \underset{\sim}{\mathbf{C}}_2, \ldots, \underset{\sim}{\mathbf{C}}_\mathbf{M}\right)}$$

$$= \frac{\left\{ \frac{\Gamma\left(\alpha_{C_m^s} + \frac{L_m - 1}{2}\right)}{\sqrt{\kappa_{C_m^s} + L_m - 1}} \frac{1}{\Psi_{m(After)}} \times \frac{\Gamma\left(\alpha_{C_{m+1}^s} + \frac{L_{m+1} + 1}{2}\right)}{\sqrt{\kappa_{C_{m+1}^s} + L_{m+1} + 1}} \frac{1}{\Psi_{m+1(After)}} \right\}}{\left\{ \frac{\Gamma\left(\alpha_{C_m^s} + \frac{L_m}{2}\right)}{\sqrt{\kappa_{C_m^s} + L_m}} \frac{1}{\Psi_{m(Before)}} \times \frac{\Gamma\left(\alpha_{C_{m+1}^s} + \frac{L_{m+1}}{2}\right)}{\sqrt{\kappa_{C_{m+1}^s} + L_{m+1}}} \frac{1}{\Psi_{m+1(Before)}} \right\}},$$

where

$$\Psi_{m(After)} = \left(\beta_{C_m^s} + \frac{\kappa_{C_m^s}\mu_{0C_m^s}^2 + \sum_{i=L_1+\ldots+L_{m-1}+1}^{L_1+\ldots+L_m-1} D_i^2 - \frac{\left(\kappa_{C_m^s}\mu_{0C_m^s} + \sum_{i=L_1+\ldots+L_{m-1}+1}^{L_1+\ldots+L_m-1} D_i\right)^2}{\kappa_{C_m^s} + L_m}}{2} \right)^{\alpha_{C_m^s} + \frac{L_m - 1}{2}}$$

$$\Psi_{m+1(After)}=\left(\beta_{C^s_{m+1}}+\frac{\kappa_{C^s_{m+1}}\mu^2_{0C^s_{m+1}}+\sum_{i=L_1+\ldots+L_m}^{L_1+\ldots+L_m+L_{m+1}}D_i^2-\frac{\left(\kappa_{C^s_{m+1}}\mu_{0C^s_{m+1}}+\sum_{i=L_1+\ldots+L_m}^{L_1+\ldots+L_m+L_{m+1}}D_i\right)^2}{\kappa_{C^s_{m+1}}+L_{m+1}}}{2}\right)^{\alpha_{C^s_{m+1}}+\frac{L_{m+1}+1}{2}}$$

$$\Psi_{m(Before)}=\left(\beta_{C^s_m}+\frac{\kappa_{C^s_m}\mu^2_{0C^s_m}+\sum_{i=L_1+\ldots+L_{m-1}+1}^{L_1+\ldots+L_m}D_i^2-\frac{\left(\kappa_{C^s_m}\mu_{0C^s_m}+\sum_{i=L_1+\ldots+L_{m-1}+1}^{L_1+\ldots+L_m}D_i\right)^2}{\kappa_{C^s_m}+L_m}}{2}\right)^{\alpha_{C^s_m}+\frac{L_m}{2}}$$

$$\Psi_{m+1(Before)}=\left(\beta_{C^s_{m+1}}+\frac{\kappa_{C^s_{m+1}}\mu^2_{0C^s_{m+1}}+\sum_{i=L_1+\ldots+L_m+1}^{L_1+\ldots+L_m+L_{m+1}}D_i^2-\frac{\left(\kappa_{C^s_{m+1}}\mu_{0C^s_{m+1}}+\sum_{i=L_1+\ldots+L_m+1}^{L_1+\ldots+L_m+L_{m+1}}D_i\right)^2}{\kappa_{C^s_{m+1}}+L_{m+1}}}{2}\right)^{\alpha_{C^s_{m+1}}+\frac{L_{m+1}}{2}}$$

$$A_{B+1}=\frac{p\left(\underset{\sim}{\mathbf{B}}_{(After)},\underset{\sim}{\mathbf{C}}_{\mathbf{1}},\underset{\sim}{\mathbf{C}}_{\mathbf{2}},\ldots,\underset{\sim}{\mathbf{C}}_{\mathbf{M}}\,\middle|\,\underset{\sim}{\mathbf{D}}\right)}{p\left(\underset{\sim}{\mathbf{B}}_{(Before)},\underset{\sim}{\mathbf{C}}_{\mathbf{1}},\underset{\sim}{\mathbf{C}}_{\mathbf{2}},\ldots,\underset{\sim}{\mathbf{C}}_{\mathbf{M}}\,\middle|\,\underset{\sim}{\mathbf{D}}\right)}$$
$$\times\frac{q\left(\underset{\sim}{\mathbf{B}}_{(Before)},\underset{\sim}{\mathbf{C}}_{\mathbf{1}},\underset{\sim}{\mathbf{C}}_{\mathbf{2}},\ldots,\underset{\sim}{\mathbf{C}}_{\mathbf{M}}\,\middle|\,\underset{\sim}{\mathbf{B}}_{(After)},\underset{\sim}{\mathbf{C}}_{\mathbf{1}},\underset{\sim}{\mathbf{C}}_{\mathbf{2}},\ldots,\underset{\sim}{\mathbf{C}}_{\mathbf{M}}\right)}{q\left(\underset{\sim}{\mathbf{B}}_{(After)},\underset{\sim}{\mathbf{C}}_{\mathbf{1}},\underset{\sim}{\mathbf{C}}_{\mathbf{2}},\ldots,\underset{\sim}{\mathbf{C}}_{\mathbf{M}}\,\middle|\,\underset{\sim}{\mathbf{B}}_{(Before)},\underset{\sim}{\mathbf{C}}_{\mathbf{1}},\underset{\sim}{\mathbf{C}}_{\mathbf{2}},\ldots,\underset{\sim}{\mathbf{C}}_{\mathbf{M}}\right)}$$

$$=\frac{\left\{\frac{\Gamma\left(\alpha_{C^s_m}+\frac{L_m+1}{2}\right)}{\sqrt{\kappa_{C^s_m}+L_m+1}}\frac{\beta_{C^s_m}^{\alpha_{C^s_m}}}{\Psi_{m(After)}}\times\frac{\Gamma\left(\alpha_{C^s_{m+1}}+\frac{L_{m+1}-1}{2}\right)}{\sqrt{\kappa_{C^s_{m+1}}+L_{m+1}-1}}\frac{\beta_{C^s_{m+1}}^{\alpha_{C^s_{m+1}}}}{\Psi_{m+1(After)}}\right\}}{\left\{\frac{\Gamma\left(\alpha_{C^s_m}+\frac{L_m}{2}\right)}{\sqrt{\kappa_{C^s_m}+L_m}}\frac{\beta_{C^*_m}^{\alpha_{C^*_m}}}{\Psi_{m(Before)}}\times\frac{\Gamma\left(\alpha_{C^s_{m+1}}+\frac{L_{m+1}}{2}\right)}{\sqrt{\kappa_{C^s_{m+1}}+L_{m+1}}}\frac{\beta_{C^s_{m+1}}^{\alpha_{C^s_{m+1}}}}{\Psi_{m+1(Before)}}\right\}},$$

where

$$\Psi_{m(After)}=\left(\beta_{C_m^s}+\frac{\kappa_{C_m^s}\mu_{0C_m^s}^2+\sum_{i=L_1+...+L_{m-1}+1}^{L_1+...+L_m+1}D_i^2-\frac{\left(\kappa_{C_m^s}\mu_{0C_m^s}+\sum_{i=L_1+...+L_{m-1}+1}^{L_1+...+L_m+1}D_i\right)^2}{\kappa_{C_m^s}+L_m}}{2}\right)^{\alpha_{C_m^s}+\frac{L_m+1}{2}}$$

$$\Psi_{m+1(After)}=\left(\beta_{C_{m+1}^s}+\frac{\kappa_{C_{m+1}^s}\mu_{0C_{m+1}^s}^2+\sum_{i=L_1+...+L_m+2}^{L_1+...+L_m+L_{m+1}}D_i^2-\frac{\left(\kappa_{C_{m+1}^s}\mu_{0C_{m+1}^s}+\sum_{i=L_1+...+L_m+2}^{L_1+...+L_m+L_{m+1}}D_i\right)^2}{\kappa_{C_{m+1}^s}+L_{m+1}}}{2}\right)^{\alpha_{C_{m+1}^s}+\frac{L_{m+1}-1}{2}}$$

$$\Psi_{m(Before)}=\left(\beta_{C_m^s}+\frac{\kappa_{C_m^s}\mu_{0C_m^s}^2+\sum_{i=L_1+...+L_{m-1}+1}^{L_1+...+L_m}D_i^2-\frac{\left(\kappa_{C_m^s}\mu_{0C_m^s}+\sum_{i=L_1+...+L_{m-1}+1}^{L_1+...+L_m}D_i\right)^2}{\kappa_{C_m^s}+L_m}}{2}\right)^{\alpha_{C_m^s}+\frac{L_m}{2}}$$

$$\Psi_{m+1(Before)}=\left(\beta_{C_{m+1}^s}+\frac{\kappa_{C_{m+1}^s}\mu_{0C_{m+1}^s}^2+\sum_{i=L_1+...+L_m+1}^{L_1+...+L_m+L_{m+1}}D_i^2-\frac{\left(\kappa_{C_{m+1}^s}\mu_{0C_{m+1}^s}+\sum_{i=L_1+...+L_m+1}^{L_1+...+L_m+L_{m+1}}D_i\right)^2}{\kappa_{C_{m+1}^s}+L_{m+1}}}{2}\right)^{\alpha_{C_{m+1}^s}+\frac{L_{m+1}}{2}}$$

Supplementary Figures

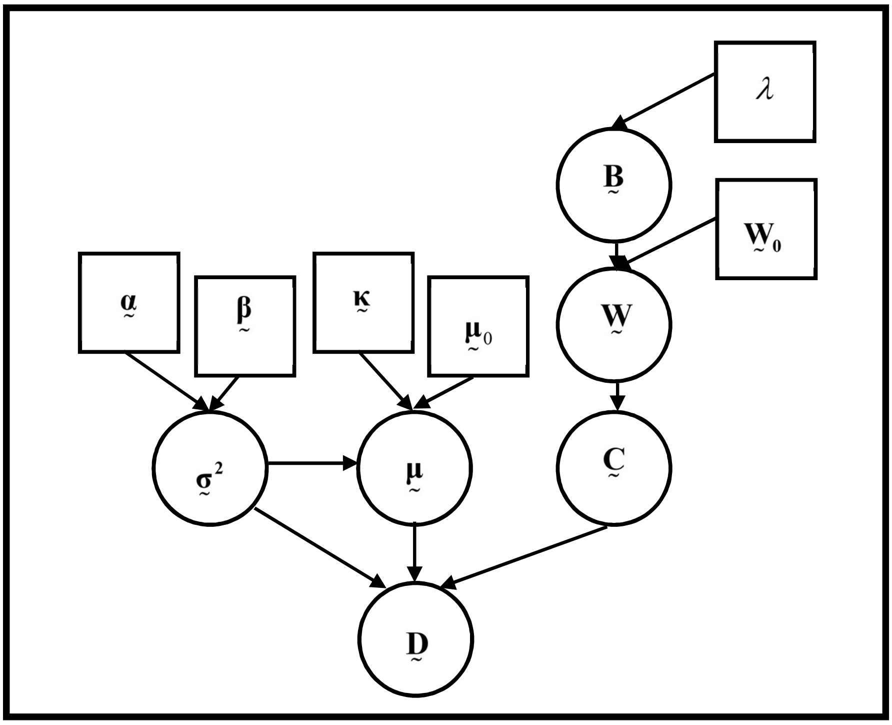

Figure S1. Variable relationships in the CONY Bayesian model.

a.

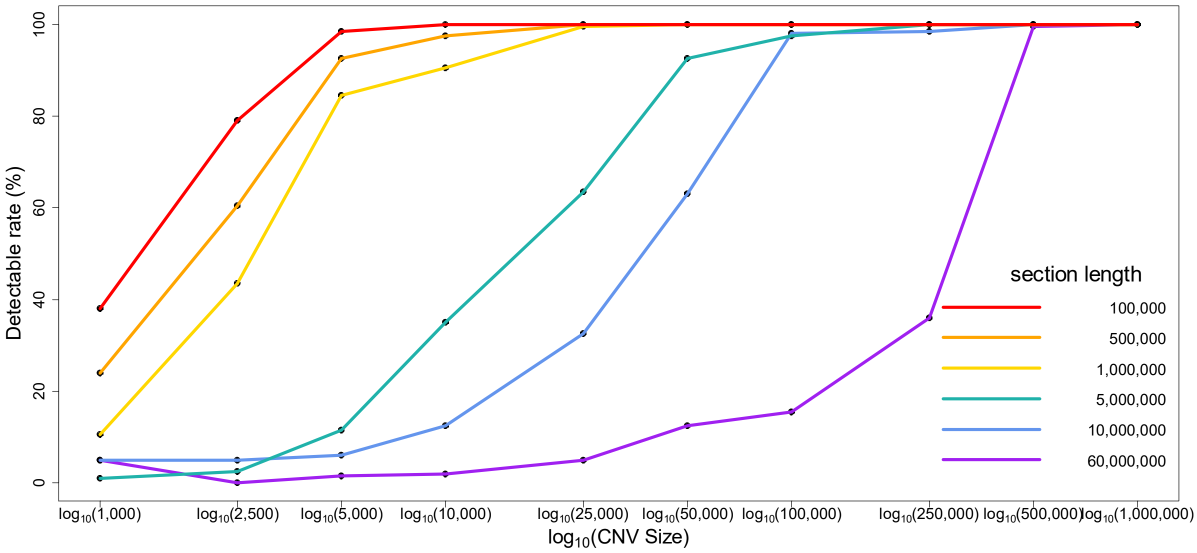

Detectable rate (%)
0
20
40
60
80
100
section length
100,000
500,000
1,000,000
5,000,000
10,000,000
60,000,000
log10(1,000)
log10(2,500)
log10(5,000)
log10(10,000)
log10(25,000)
log10(50,000)
log10(100,000)
log10(250,000)
log10(500,000)
log10(1,000,000)
log10(CNV Size)

b.

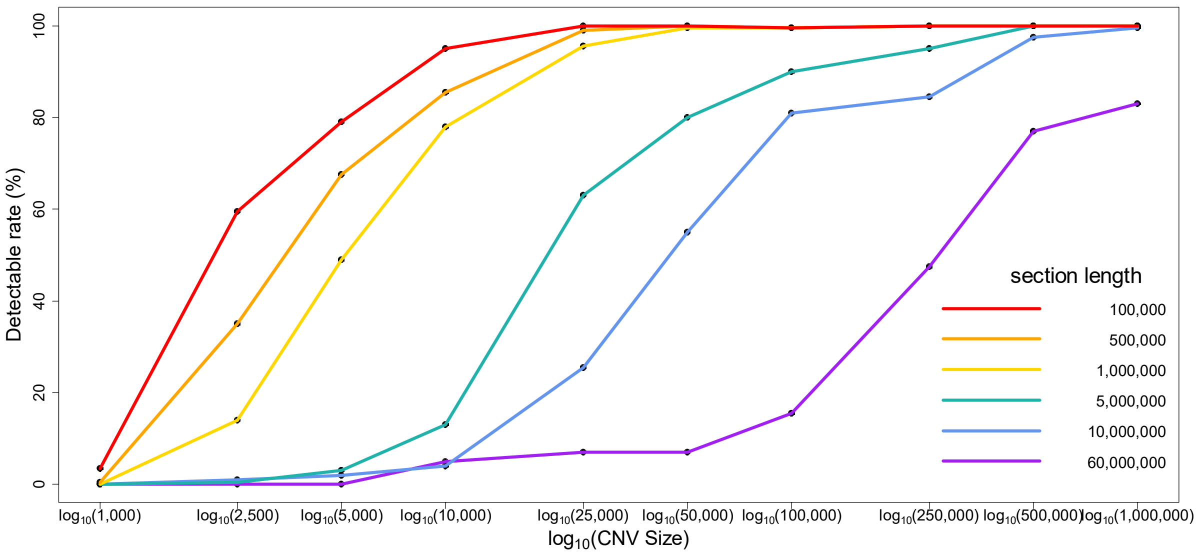

Detectable rate (%)
0
20
40
60
80
100
section length
100,000
500,000
1,000,000
5,000,000
10,000,000
60,000,000
log10(1,000)
log10(2,500)
log10(5,000)
log10(10,000)
log10(25,000)
log10(50,000)
log10(100,000)
log10(250,000)
log10(500,000)
log10(1,000,000)
log10(CNV Size)

c.

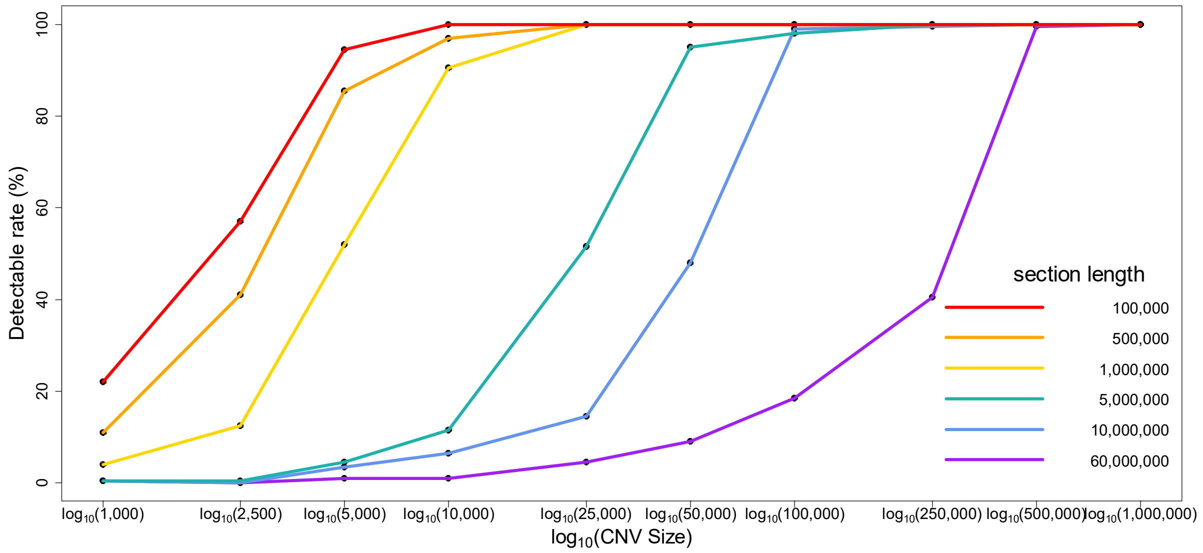

d.

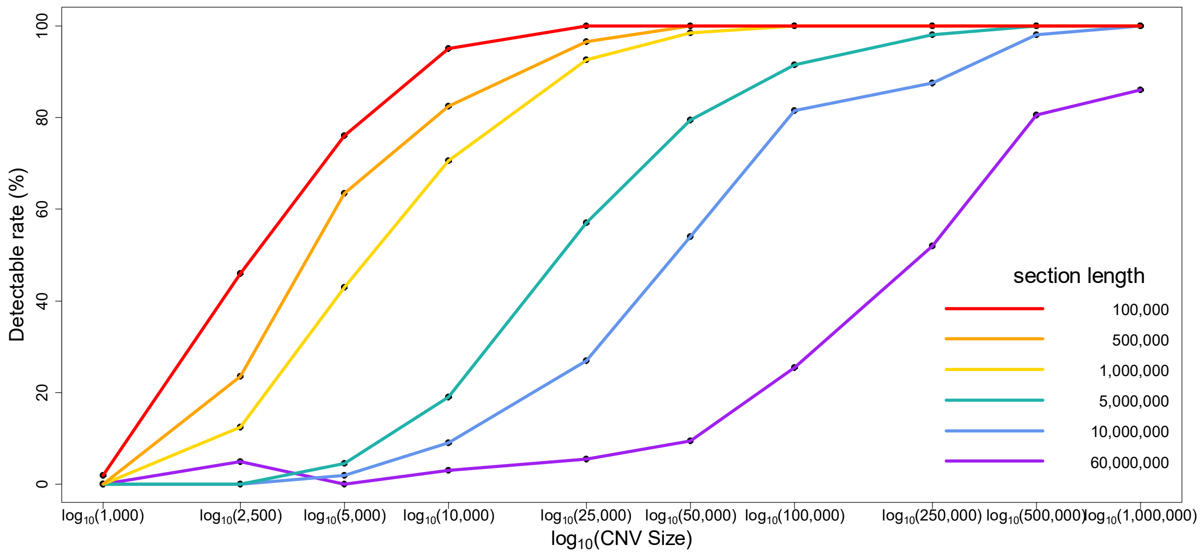

Figure S2. Effects of analytic section length on the CNV detection rate. Results of **a.** the detection of copy losses in the single-sample analysis, **b.** the detection of copy gains in the single-sample analysis, **c.** the detection of copy losses in the paired-samples analysis, and **d.** the detection of copy gains in the paired-samples analysis.

Supplementary Tables

Table S1. Performance of CNV detection in the low- and high-coverage experimental data.

(a) Low-coverage data

Sample(s)	Algorithm	CNV bases				CNV regions	
		bp[a]	Recall	FPR	Precision	Region[b]	Detection rate
**Single-sample analysis** (HG00419)	CONY	1,054,535	72.37%	6.56%	6.56%	63	79.75%
	CNVnator	433,261	29.73%	0.50%	38.03%	47	59.49%
	FREEC	231,270	15.87%	1.07%	9.34%	23	29.11%
	rdxplorer[c]	-	-	-	-	-	-
	**DGV**	**1,457,096**				**79**	
**Paired-samples analysis** (Case:HG00419/ Control:HG01595)	CONY	194,133	63.41%	4.44%	1.81%	16	43.24%
	CNVSeq	158,142	51.66%	0.41%	16.68%	36	97.30%
	FREEC	0	0.00%	0.00%	8.43%	0	0.00%
	**DGV**	**306,145**				**37**	

Sample(s)	Algorithm	CNV bases				CNV regions	
		bp[a]	Recall	FPR	Precision	Region[b]	Detection rate
**Single-sample analysis** (HG01595)	CONY	1,018,097	78.62%	5.95%	7.03%	52	81.25%
	CNVnator	411,884	31.81%	1.54%	11.51%	29	45.31%
	FREEC	125,456	9.69%	0.48%	11.43%	8	12.50%
	rdxplorer[c]	-	-	-	-	-	-
	**DGV**	**1,295,026**				**64**	
**Paired-samples analysis** (Case:HG01595/ Control:HG00419)	CONY	232,383	75.91%	3.77%	2.58%	20	54.05%
	CNVSeq	158,142	51.66%	0.41%	16.64%	36	97.30%
	FREEC	0	0.00%	0.00%	0.24%	0	0.00%
	**DGV**	**306,145**				**37**	

[a] The number of CNV basepairs in the DGV that are also identified by the algorithm
[b] The number of CNV regions in the DGV that have any position identified as a CNV via the algorithm
[c] rdxplorer does not support genome GRCh38/hg38

(b) High-coverage data

Sample(s)	Algorithm	CNV bases				CNV regions	
		bp[a]	Recall	FPR	Precision	Region[b]	Detection rate
**Single-sample analysis** (HG00419)	CONY	964,604	66.20%	7.69%	5.05%	65	82.28%
	CNVnator	618,995	42.48%	2.77%	9.49%	54	68.35%
	FREEC	593,266	40.72%	2.51%	10.08%	26	32.91%
	rdxplorer[c]	-	-	-	-	-	-
	**DGV**	**1,457,096**				**79**	
**Paired-samples analysis** (Case:HG00419/ Control:HG01595)	CONY	230,088	75.16%	5.61%	1.68%	21	56.76%
	CNVSeq	177,056	57.83%	1.33%	5.71%	36	97.30%
	FREEC	159,990	52.26%	3.90%	1.71%	5	13.51%
	**DGV**	**306,145**				**37**	

Sample(s)	Algorithm	CNV bases				CNV regions	
		bp[a]	Recall	FPR	Precision	Region[b]	Detection rate
**Single-sample analysis** (HG01595)	CONY	776,547	59.96%	6.34%	5.00%	51	79.69%
	CNVnator	359,729	27.78%	1.11%	14.04%	39	60.94%
	FREEC	203,488	15.71%	0.95%	9.23%	9	14.06%
	rdxplorer[c]	-	-	-	-	-	-
	**DGV**	**1,295,026**				**64**	
**Paired-samples analysis** (Case:HG01595/ Control:HG00419)	CONY	251,031	82.00%	6.46%	1.58%	21	56.76%
	CNVSeq	177,056	57.83%	1.33%	5.71%	36	97.30%
	FREEC	187,848	61.36%	0.85%	9.54%	6	16.22%
	**DGV**	**306,145**				**37**	

[a] The number of CNV basepairs in the DGV that are also identified by the algorithm
[b] The number of CNV regions in the DGV that have any position identified as a CNV via the algorithm
[c] rdxplorer does not support genome GRCh38/hg38

Table S2. CNV detection base accuracy with various section lengths in the RJMCMC algorithm.

Section length (Mb)	(%) Overall accuracy	Single-sample analysis				Paired-samples analysis				
		Copy loss		Copy gain		Overall	Copy loss		Copy gain	
		Recall	FPR	Recall	FPR	accuracy	Recall	FPR	Recall	FPR
**60**	96.03	76.03	0.07	64.18	0.002	96.46	76.40	0.004	69.55	0.01
**10**	98.84	95.24	0.14	88.87	0.003	99.05	94.31	0.01	91.54	0.02
**5**	99.19	97.42	0.25	93.48	0.007	99.48	96.88	0.01	95.64	0.02
**1**	99.26	99.28	0.60	97.48	0.006	99.82	99.24	0.01	98.53	0.02
**0.5**	99.21	99.44	0.70	97.85	0.004	99.86	99.50	0.02	98.82	0.01
**0.1**	99.05	99.41	0.87	97.76	0.004	99.85	99.54	0.05	99.01	0.01

Table S3. CNV detection performance with different read coverage and depth estimation approaches.

(%)	Coverage	Depth estimating method	Detection rate of each CNV size (bp)										Base accuracy	
			1K	2.5K	5K	10K	25K	50K	100K	250K	500K	1M	Recall	FPR
Copy loss	2.2 X	summing-up	24.0	60.5	92.5	97.5	100.0	100.0	100.0	100.0	100.0	100.0	99.44	0.70
		representative-position	2.0	19.5	71.0	93.0	99.5	100.0	100.0	100.0	100.0	100.0	99.12	0.28
	22 X	summing-up	63.0	94.5	99.0	100.0	100.0	100.0	100.0	100.0	100.0	100.0	99.18	1.24
		representative-position	65.0	95.0	99.5	100.0	100.0	100.0	100.0	100.0	100.0	100.0	99.30	1.20
Copy gain	2.2 X	summing-up	0.5	35.0	67.5	85.5	99.0	100.0	99.5	100.0	100.0	100.0	97.85	0.004
		representative-position	0.0	18.5	56.5	71.0	95.0	99.5	99.5	100.0	100.0	100.0	97.79	0.004
	22 X	summing-up	23.5	79.0	91.5	98.0	100.0	100.0	99.5	100.0	100.0	100.0	98.45	0.13
		representative-position	40.5	79.5	90.5	97.5	100.0	100.0	99.5	100.0	100.0	100.0	98.57	0.06
